# Exploring the mechanisms of flavor formation and polyphenolic changes in hop-infused sourdough bread affected by hop varieties and soaking methods

**DOI:** 10.1016/j.fochx.2025.102512

**Published:** 2025-05-02

**Authors:** Chunyuan Ping, Bian Li, Yueyue Gao, Xiang Li, Fu Wang

**Affiliations:** a Food Science and Engineering, Sichuan Tourism University, Chengdu, Sichuan Province 610100, China; b School of Food Science and Technology, Henan Institute of Science and Technology, Xinxiang, Henan Province, 453003, China

**Keywords:** Hop bread, Volatile organic compounds, Polyphenols, Bionic sensory, HS-GC-IMS

## Abstract

*Humulus lupulus L.* (hops) is globally recognized in food production as a flavor enhancer and flour improver. Here, different hop-soaking methods (cold and boiling water) and varieties (Tsingtao and Saaz) were investigated to determine how these influence the flavor profile of hop-based sourdough bread. Headspace gas chromatography-ion mobility spectrometry (HS-GC-IMS), ultra-performance liquid chromatography-orbitrap-mass spectrometry, automatic amino acid analysis, and bionic sensors were used to analyze volatile organic compounds (VOCs), amino acids, polyphenols, and sensory attributes. The results showed significant differences in flavor compounds between hop varieties and soaking methods. Cold-water soaked of Tsingtao hops had enhanced aroma intensity, particularly for ketones and aldehydes, while boiling water treatment increased bitter acids and polyphenols in Saaz hops. Pearson and Mantel's tests showed strong correlations between amino acids, VOCs, and polyphenols, with *E*-nose and E-tongue results aligning closely with sensory scores. These findings provide a theoretical foundation for optimizing hop bread production.

## Highlights

• Hop soaking methods altered volatile profiles, shaping unique flavor characteristics.
• Cold-soaked Tsingtao hops enhanced fruity and floral aroma, improving overall sensory scores.
• Tsingtao hops reduced amino acids, promoting the formation of key volatile precursors.
• Boiled Saaz hops increased polyphenols, boosting antioxidant capacity and bitterness.

## Graphical abstract

**Figure:**
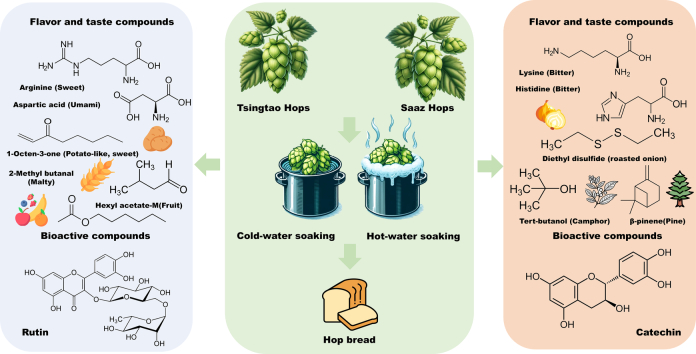

## 1 Introduction

*Humulus lupulus L.* (hops) is valued globally in food production as a flavor enhancer, flour improver, and natural preservative, exhibiting diverse biological activities (Sun et al., 2022). Hops possess complex phytochemical profiles and significant pharmacological potential, with therapeutic effects linked to their modulation of cancer-related pathways, including apoptosis regulation, extracellular matrix modulation, angiogenesis inhibition, potential cancer-preventive agent and oxidative damage reduction (Girisa et al., 2021; Kowalska et al., 2022). For centuries, hops have been utilized in bread-making, with demand increasing. Research reveals that hop constituents affect colloidal and biochemical processes during bread-making (Knez Hrnčič et al., 2019). Chaplygina et al. (2020) reported that hop sourdough significantly reduces the risk of pathogenic microflora, including *Bacillus subtilis* and mold fungi, in bread products.

Additionally, hop-sourdough-fermented bread exhibits sensory and physicochemical properties comparable to commercially yeasted bread. Zhemela et al. (2020) revealed that hop-infused dough significantly enhances the organoleptic properties of bread, imparting desirable aroma and taste. Hops serve as a natural preservative, inhibiting harmful microorganisms during the storage and transport of bread. Polyphenols play a critical role in this preservation mechanism, with compounds like flavonoids, phenolic acids, and tannins demonstrating substantial antimicrobial properties. These polyphenolic compounds interact with microbial cell membranes, disrupt protein structures, and inhibit enzymatic activities, effectively preventing microbial growth and extending bread shelf life (Knez Hrnčič et al., 2019). This reduces reliance on chemical preservatives and mitigates associated health risks (Betancur et al., 2023).

Flavor is a crucial factor in purchasing decisions for consumers, comprising aroma and taste, with aroma being one of the first characteristics perceived by customers when purchasing bread (Chen et al., 2022). The flavor profiles of hops are affected by genetic factors, environmental conditions, and varietal differences, particularly from European and Asian varieties, with significant contributions from hops produced in the Czech Republic and China (Kubeš, 2021). Regional variations and production practices significantly influence hop flavor, affecting consumer preferences and market trends. Hop bread is prized for its distinctive flavor and bioactive properties.

The hop soaking method is crucial in preparing hop sourdough, which affects fermentation by imparting flavors, inhibiting pathogens, and enhancing organoleptic qualities. Different soaking techniques affect hop compound releasing, altering aroma profiles and microbial communities in sourdough. Additionally, compounds including esters, diacetyl, organic acids, and higher alcohols from branched-chain amino acids through the Ehrlich pathway are influenced by the soaking method (Gänzle & Zheng, 2019; Suo et al., 2020). Furthermore, the chemical constituents of hop sourdough engage in complex reactions, intensifying bread flavor and generating precursors that transform during baking (Semko et al., 2024).

Over 1000 compounds have been identified in hops using advanced analytical techniques (Ou et al., 2024). These compounds include bitter compounds such as α-acids, β-acids, polyphenols, and humulones, alongside aromatic compounds categorized into the following three main groups: hydrocarbons, oxygen-, and sulfur-containing compounds (Henning et al., 2021). Gas chromatography-ion mobility spectrometry (GC-IMS) vaporizes sample molecules at atmospheric or near-atmospheric pressure, ionizing them to produce product ions at the ionization source. GC-IMS contains rapid response time and efficient detection, which are especially beneficial for trace analysis—detecting components at concentrations below one part per million (Wang et al., 2020). Individual factors can influence sensory evaluation, potentially compromising the consistency of the results. Conversely, the electronic nose (*E*-nose) and tongue (*E*-tongue), equipped with sensitive sensors, were used to capture odor and taste signals, reflecting characteristic food flavor information.

Despite the increasing interest in hop bread, research has primarily concentrated on hops in beer production, leaving a significant gap in understanding how regional hop varieties and soaking methods influence bread flavor profiles. Understanding these complex flavor variations is essential for optimizing hop bread production. To address this gap, this study employed HS-GC-IMS, *E*-nose, and E-tongue, along with multivariate statistical analysis. Therefore, this study aims to investigate the flavor compound composition of hop bread prepared using cold and boiled soak methods and to compare the characteristic flavor variations between Tsingtao (China) and Saaz (Czech) varieties. This study could provide a theoretical foundation for enhancing hop bread production.

## 2 Material and methods

### 2.1 Material and reagents

Tsingtao Flower hop, harvested in July 2023, was obtained from Tsingtao Brewery Company Co., Ltd. (Tsingtao, Shandong, China). Czech Saaz hop, also harvested in July 2023, was obtained from Stanau Hops Co., Ltd. (Zhuhai, Guangdong province, China). Sulfosalicylic acid and N-ketones (C4-C9) were purchased from Sinopharm Group Co. Ltd. (China). Yeast was sourced from Angel Yeast Co., Ltd. (China), while methanol, acetonitrile, and acetic acid were procured from ANPEL Laboratories Technologies Inc. (Shanghai, China). All standards of polyphenol were purchased from Sigma-Aldrich (Louis, MO, USA, ≥98 % purity). All solvents used were of liquid chromatography-mass spectrometry (LC-MS) grade. Ultrapure water was prepared using a Milli-Q water purification system (Millipore, Bedford, MA, USA). All samples were vacuum-sealed and stored at −20 °C in a dark environment to maintain their integrity.

#### 2.1.1 Sample preparation

(1) Preparation of sourdough starter: A mixture of 200 g of water, 300 g of wheat flour, and 2 g of sugar underwent fermentation for 24 h at ambient temperature (21–23 °C). Subsequently, 100 g of wheat flour and 2 g of sugar were added for a second 24-h fermentation. In the third and fourth fermentation cycles, 150 g of wheat flour and 3 g of sugar were incorporated at the beginning of each 24-h period.

(2) Control dough preparation: The control dough consisted of a mixture of 550 g of wheat flour, 910 g of sourdough starter, 12 g of salt, 100 g of sugar, 5 g of active dry yeast, and 30 g of distilled water. Fermentation occurred at 36 °C with 85 % relative humidity for 60 min, followed by baking at 230 °C for 30 min. This sample was labeled “K.”

(3) Preparation of hop sourdough starter: Tsingtao Flower and Saaz hops were ground to a 60-mesh size using a laboratory grinder (LB-10EGG, Laboratory Scientific, Wilmington, USA). Two grams of hop powder were soaked in 200 g of cold (4 °C) or boiling (100 °C) distilled water for 2 h. The mixture was then filtered, and the resulting hop filtrate was utilized for sourdough preparation.

(4) Hop sourdough preparation: For the hop sourdough, 200 g of hop filtrate replaced distilled water, achieving a moisture content of 57 %. The remaining ingredients and procedures matched those used for the control samples. Bread made with Tsingtao Flower hop pellets soaked in boiling water was labeled “GK,” while those made with cold water extract were labeled “GL.” Similarly, Saaz hop pellets soaked in boiling water were labeled “JK,” and those with cold water extract were designated “JL.”

### 2.2 Sensory evaluation

A panel of ten graduate students specializing in Food Science, consisting of four males and six females aged between 20 and 30 years, participated in the sensory study. All panelists had prior experience with sensory analysis through coursework and research activities. Additionally, none of the participants reported dietary restrictions or preferences that could impact their sensory perceptions, ensuring unbiased and consistent evaluation. Before the assessment, the panelists completed an intensive training program lasting over 1 month to enhance their proficiency in descriptive sensory analysis techniques for hop bread. Rigorous screening tests ensured that each panelist exhibited optimal olfactory and gustatory sensitivity, with no indications of oral or nasal health issues. A quantitative descriptive analysis was conducted to evaluate the bread samples, concentrating on parameters, such as appearance texture, taste, and aroma. A predefined lexicon, including reference materials and intensity scales (Table S1), was used to standardize the evaluations. To prevent sensory fatigue, evaluations were conducted in triplicate with 25-min breaks between sessions. Bread samples weighing 25 ± 0.5 g were presented randomly on sterile disposable plates, each labeled with unique three-digit codes. The evaluations occurred in individual sensory booths maintained under controlled environmental conditions (temperature: 23 ± 1 °C; relative humidity: 50 ± 5 %; illumination: 750 lx) to ensure consistency and accuracy. Each sample was evaluated in triplicate by all panelists to ensure the reliability and consistency of the sensory results.

All participants provided informed consent, confirming their understanding of the study and voluntary participation.

### 2.3 pH analysis

The pH values of the dough and bread were measured using an automatic pH meter (LC-PH-3S-pH meter, Shanghai Lichen Instrument Technology Co., Ltd., Shanghai, China). Before each measurement, the probe was rinsed with ultrapure water, and the pH meter was calibrated at three points with a buffer solution before use. Sample measurements were conducted in triplicates.

### 2.4 Polyphenol analysis

Polyphenol detection followed the methodology outlined by Zhang et al. (2019). (1) Sample preparation: Samples were treated with 0.5 mL of 80 % methanol. Ultrasonic extraction was performed at 40 kHz, 600 W for 30 min, followed by centrifugation at 12,000 rpm for 10 min and the supernatant was collected. This procedure was conducted in triplicates, and the supernatant was combined.

(2) Ultra-high performance liquid chromatography (UHPLC) system parameters: The extracted samples were analyzed using a UHPLC-Orbitrap-MS system (Vanquish UPLC, QE mass spectrometer, Thermo Fisher Scientific, Waltham, MA, USA). The UHPLC-specific settings included a Waters HSS T3 column (50 × 2.1 mm, 1.8 μm) with a column temperature of 40 °C, flow rate of 0.3 mL/min, and injection volume of 2 μL. The solvent system comprised water (0.1 % formic acid) and acetonitrile (0.1 % formic acid). The gradient program was as follows: 90:10 *V*/V at 0 min, maintained at 90:10 V/V for 2.0 min, adjusted to 40:60 V/V for 6.0 min, held at 40:60 V/V for 9.0 min, returned to 90:10 V/V at 9.1 min, and concluded at 12.0 min.

(3) Mass spectrometry: High-resolution mass spectrometry data were acquired using a Q Exactive Hybrid Q-Orbitrap mass spectrometer (Thermo Fisher Scientific) equipped with a heated electrospray ionization (ESI) source. The Full MS/MS acquisition mode was employed, with ESI parameters set to a spray voltage of −2.8 kV, sheath gas pressure at 40 arbitrary units (arb), auxiliary gas pressure at 10 arb, capillary temperature of 320 °C, and auxiliary gas heater temperature of 350 °C. Data were acquired using a Q-Exactive mass spectrometer and processed with Xcalibur 4.1 software (Thermo Fisher Scientific). Quantitative data were analyzed using TraceFinder™ 4.1 Clinical (Thermo Fisher Scientific). The experiment was conducted in triplicate to ensure the reliability and consistency of the results.

### 2.5 Analysis of *E*-tongue

The E-tongue system (Alpha MOS α-ASTREE, Toulouse, France) was employed to evaluate the taste attributes of hop bread samples. This system incorporates five distinct sensors designed to detect sourness (AHS), saltiness (CTS), umami (NMS), sweetness (ANS), and bitterness (SCS). Each sensor contains a lipid/polymer membrane that interacts with taste compounds via electrostatic and hydrophobic interactions, generating specific electrical potential patterns that correspond to different taste qualities. To prepare the bread samples, 25 g of crumb was homogenized in 150 g of ultrapure water and subjected to sonication at 60 kHz for 30 min. The homogenized solution was then filtered before analysis. Each sample underwent a 120 s analysis, with sensors thoroughly rinsed between measurements to prevent cross-contamination. Multiple replicates were conducted to ensure the statistical robustness and accuracy of the results.

### 2.6 Determination of free amino acids (FAA)

FAA was determined as per the method outlined by Qiao et al. (2024). Quantification of amino acids was conducted using an automatic amino acid analyzer (S433D, Sykam, Munich, Germany). Samples were prepared using the sulfosalicylic acid hydrolysis method. Breadcrumbs weighing 25 g were homogenized in ultrapure water and then mixed with an equal mass of a 7 % sulfosalicylic acid solution. The mixture underwent sonication (SG900HPT, Xinling Instrument Equipment Co., Ltd., Zhengzhou City, China) at 60 kHz for 30 min to ensure complete hydrolysis, followed by filtration. The resulting filtrate was centrifuged (TGL-18, Shuke Instrument Co., Ltd., Chengdu City, China) at 15,000 rpm for 15 min to eliminate particulates. A supernatant of 1 mL was further filtered through a 0.22 μm microporous membrane (Sigma Aldrich Trading Co., Ltd., Shanghai, China) and transferred to a vial for subsequent analysis. Amino acid separation and quantification were conducted using a PEEK column (4.6 mm × 150 mm, 7 μm particle size, 10 % cross-linking) with a temperature gradient ranging from 20 °C–99 °C. The reactor temperature was maintained at 130 °C throughout the analysis. Detection occurred at wavelengths of 570 and 440 nm. The analysis lasted 60 min, with a ninhydrin reagent flow rate of 0.25 mL/min and an injection volume of 40 μL. Free amino acids were conducted in triplicate to ensure the reliability and consistency of the results.

### 2.7 Analysis of *E*-nose

The odor profile was analyzed using a Fox 4000 E-nose system (Alpha MOS, Toulouse) equipped with 18 sensor chambers. Table S2 presents detailed specifications of the sensors. Breadcrumbs weighing 2 g were placed in a sealed 10 mL glass vial and heated to 55 °C for 3 min to generate headspace volatiles. The detection phase lasted 150 s, during which the sensors recorded stable signals. After each measurement, the system was reset by flushing the gas path with zero gas (air passed through activated carbon) for 360 s to eliminate residual odor. Sensor readings were captured every second and saved after the process. Five replicates were performed for each sample to ensure reliability and consistency in odor characterization and three stable datasets were selected from the ten for further analysis.

### 2.8 Analysis of headspace gas chromatography-ion mobility spectrometry (HS-GC-IMS)

The bread samples were analyzed for volatile organic compounds (VOCs) using a thermal desorption HS-GC-IMS system (FlavourSpec®, G.A.S., Germany) set at 60 °C. VOC separation was achieved with an MXT-WAX capillary column (15 m × 0.53 mm i.d., 1 μm film thickness). The analytes were ionized in the IMS ionization chamber, using tritium radioactive source, with the chamber temperature maintained at 80 °C. The ionized compounds traversed 53 mm drift tube under an electric field strength of 550 V/cm, which was also held at 80 °C. High-purity nitrogen (99.999 %) functioned as the carrier gas in the GC and as the drift gas in the IMS, flowing against the analyte ions at a rate of 150 mL/min. The IMS system operated in positive ionization mode. Each bread type (K, GL, GK, JL, and JK) had 5 g of breadcrumbs placed in a 20 mL headspace vial. The samples were equilibrated at 70 °C for 25 min while being agitated at 600 rpm. Following equilibration, 200 μL of headspace was injected into the GC-IMS system through a heated syringe (80 °C) and a temperature-controlled injection port. Three replicate analyses ensured reproducibility. The retention indices of the identified VOCs were determined using n-ketones (C4-C9) as external standards. Compounds were identified by comparing retention indices and drift times with reference databases from the NIST and IMS libraries. VOCs were quantified in a semi-quantitative manner by calculating the integrated peak volumes (signal intensities) from GC-IMS spectra using VOCal software (G.A.S., version 0.4.03).

#### 2.8.1 Evaluation of key compounds

The relative odor activity value (ROAV) method was employed to identify key VOCs in bread samples (Ping et al., 2024). VOCs were quantified using the following formula:

$$\text{ROAV}\approx 100\times \frac{C{\%}_{X}}{C\%\mathrm{stan}}\times \frac{T_{\mathrm{stan}}}{T_{X}}$$

where C%*stan* and T*stan* represent the percentage (%) and threshold (μg/kg) of the components that significantly contribute to the flavor of bread samples. C%_*X*_ and T_*X*_ denote the percentage and threshold (μg/kg) of each VOC. VOCs with ROAV >1 were identified as key aroma compounds, whereas those with 0.1 ≤ ROAV/OAV ≤ 1 were identified as compounds with a modifying effect on the sensory characteristics of bread samples.

### 2.9 Data processing and statistical analysis

Principal component analysis (PCA) was conducted using Origin 2024b software (OriginLab Corporation, Northampton, MA, USA). A one-way analysis of variance (ANOVA) was conducted using SPSS 24.0 (IBM Corporation, Armonk, NY, USA) at a significance level of *P* < 0.05. Orthogonal partial least squares discriminant analysis (OPLS-DA) was performed with SIMCA software (version 14.1, Umetrics, Umeå, Sweden). Spectral comparisons were facilitated by the Reporter plugin in GC-IMS software (FlavourSpec®, G.A.S.), generating characteristic spectra through the Gallery Plot plugin. The structure of VOCs was visualized with ChemDraw 20.0 (PerkinElmer), and quantitative analysis of VOCs was conducted using the VOCal software (Version 0.4.03, G.A.S.). Pearson's and Mantel's tests were conducted using Chiplot (https://www. chiplot. online/#Networkplot).

## 3 Results and discussion

### 3.1 Sensory analysis

**Fig. 1:**
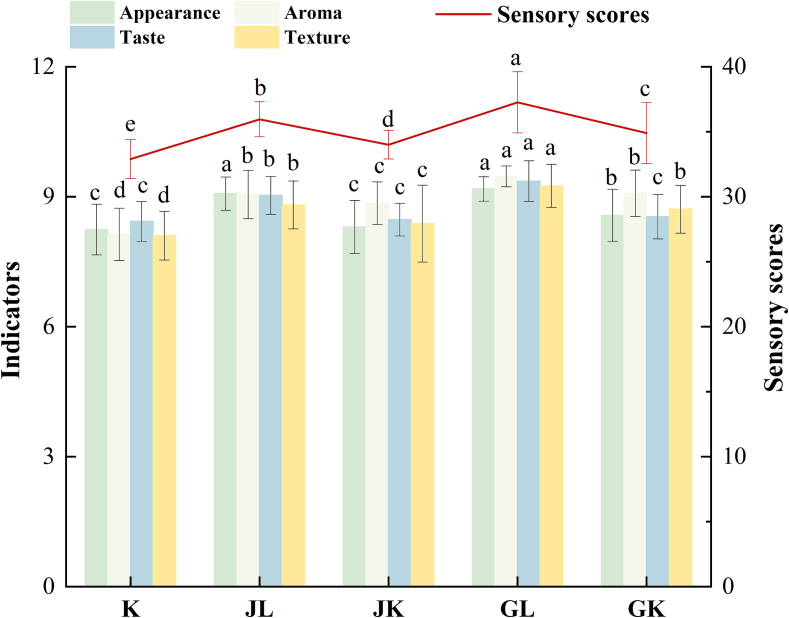
Sensory evaluation of bread prepared with various hop varieties soaked in cold and hot water. Note: The control sample was designated as “K”; Tsingtao Flower hop pellets soaked in boiling water was labeled “GK”; Tsingtao Flower hop pellets soaked in cold water was labeled “GL”; Saaz hop pellets soaked in boiling water was labeled “JK”; Saaz hop pellets soaked in cold water was labeled “JL”.

As shown in Fig. 1 and Table S3, there was a significant increase (*P* < 0.05) in bread sensory scores with hop addition, independent of the soaking method used. Li et al. (2023) reported that lupulones from hops effectively enhance food sensory perception and improve aroma detection and overall quality. The addition of hops significantly improves the organoleptic properties of bread, with the soaking method influencing the extent. Expert panel results indicated that cold steeping significantly improved all sensory attributes (*P* < 0.05), while boiled soaking only enhanced aroma and texture, there were no significant improvements in taste. Furthermore, hops origin influenced bread quality; Saaz hops significantly enhanced appearance (*P* < 0.05), while Tsingtao hops improved overall quality, particularly aroma (*P* < 0.05). Aroma compounds in hops are primarily influenced by genetic factors, with varietal differences significantly affecting product quality (Guimarães et al., 2021). Therefore, investigating regional and varietal hop differences is essential for optimizing hops-infused bread production. It is worth noting that the sensory evaluation was based on a limited panel of trained assessors. Future studies will broaden this by including a larger and more diverse group, incorporating both experts and general consumers.

### 3.2 pH analysis

pH significantly influenced dough properties and bread quality. The optimal pH range (5–7) activates enzymes in the dough, including proteases, promoting protein degradation and gluten development, resulting in a cohesive and elastic structure (Feng et al., 2022). Table 1 illustrates pH changes (*P* < 0.05) in hop sourdough and bread. The pH of sourdough infused with hops varies with hop variety and soaking method; cold water soaking raised pH, with significant effects noted in boiling water samples. This trend was also hops-dependent; Tsingtao hops exhibited a significantly higher pH than that of Saaz hops, with a statistically significant difference (*P* < 0.05). Post-baking, dough pH increased, aligning with the sourdough trend while remaining within the ideal range, consistent with sensory evaluations. Bread made with cold water-soaked Tsingtao hops exhibited the highest pH increase, which may be attributed to the rich bioactive compounds in hops—α-acids, β-acids, and essential oils—that effectively inhibit the growth of lactic acid and acetic acid bacteria during dough fermentation (Maino et al., 2024). This antimicrobial action prevents excessive acid production while extending dough shelf life by suppressing bacterial proliferation and spoilage (Raj et al., 2022; Sun et al., 2022).

**Table 1:** The pH values of hop bread and sourdough.

Sample	Dough	Bread
K	5.00 ± 0.22^c^	5.20 ± 0.17^e^
GL	5.10 ± 0.13^b^	5.55 ± 0.04^a^
GK	5.23 ± 0.06^a^	5.34 ± 0.32^c^
JL	4.80 ± 0.21^e^	5.40 ± 0.12^b^
JK	4.90 ± 0.09^d^	5.30 ± 0.27^d^

Note: a, b, c Means with different letters with in a row differ significantly (*P* < 0.05). ±: standard deviation. The control sample was designated as “K”; Tsingtao Flower hop pellets soaked in boiling water was labeled “GK”; Tsingtao Flower hop pellets soaked in cold water was labeled “GL”; Saaz hop pellets soaked in boiling water was labeled “JK”; Saaz hop pellets soaked in cold water was labeled “JL”. pH was conducted in triplicate to ensure the reliability and consistency of the results.

### 3.3 Polyphenol analysis

Owing to their bioactive polyphenolic content, hops have the potential to improve the quality of low-quality bread products (Carbone & Macchioni, 2025). Polyphenols offer various health benefits, including antioxidant, antibacterial, and antitumor properties (Kowalska et al., 2022). The antioxidant activity of hop polyphenols is four times greater than that of vitamin C, suggesting they may delay the aging process in organisms (Kowalska et al., 2022). Table 2 lists 38 polyphenolic compounds detected in hops bread, with quercetin, rutin, benzoic acid, kaempferol, and catechin as the most abundant. The contributions of these compounds varied with different soaking methods and hop varieties. Di Domenico et al. (2020) reported that hops contain significant amounts of quercetin and rutin, with their concentrations influenced by the extraction method used. The hydroxyl groups of quercetin provide antioxidant and anti-apoptotic effects on cancer cell lines, making it a particularly significant flavonoid (Slika et al., 2022). Owing to varietal differences, Tsingtao hops contained significantly higher quercetin levels than those in Saaz hops, irrespective of whether they were soaked in cold or hot water. Furthermore, soaking methods can cause variations in polyphenol content; cold water soaking for Tsingtao hops yielded higher levels than those of hot water soaking, while the opposite was true for Saaz hops.

**Table 2:** Characterization of phenolic compounds in different hop breads by UPLC-Orbitrap-MS.

Number	Compounds	Expected Mass (*m*/*z*)	Retention time	Curve equation	R^2^	RSD%	K	GL	GK	JL	JK
1	Quercetin	301.03538	7.030	Y = 3.855e5X	0.9975	2.0705	2.33 ± 0.15^d^	359.95 ± 13.17^a^	339.77 ± 22.02^ab^	294.01 ± 11.53^c^	333.51 ± 8.16^b^
2	Rutin	609.14611	5.570	Y = 1.934e5X	0.9983	3.7792	4.5 ± 0.3^d^	283.8 ± 10.38^b^	432.59 ± 28.04^a^	35.27 ± 1.11^d^	72.04 ± 1.76^c^
3	Trans-Ferulic acid	193.05063	5.630	Y = 2.684e5X	0.9965	1.9631	104.93 ± 6.97^d^	130.29 ± 4.77^c^	142.32 ± 9.22^ab^	139.54 ± 4.39^bc^	151.73 ± 3.71^a^
4	Benzoic acid	121.0295	5.980	Y = 1.205e5X	0.9951	2.1675	92.91 ± 6.17^c^	126.34 ± 4.62^b^	132.02 ± 8.56^ab^	140.49 ± 4.41^a^	128.6 ± 3.15^b^
5	Kaempferol	285.04046	7.610	Y = 6.458e5X	0.997	1.5986	2.82 ± 0.19^c^	74.62 ± 2.73^b^	65.91 ± 4.27^b^	187.36 ± 5.89^c^	179.66 ± 11.11^c^
6	Catechin	289.07176	3.940	Y = 2.267e5X	0.9985	0.1720	3.96 ± 0.26^d^	27.78 ± 1.02^c^	35.34 ± 2.29^c^	154.18 ± 4.85^bc^	217.27 ± 13.44^a^
7	Quercetin 3-β-D-glucoside	463.0882	5.710	Y = 2.617e5X	0.9997	3.8123	16.69 ± 1.11^e^	41.58 ± 1.52^b^	52.86 ± 3.43^a^	20.18 ± 1.25^d^	35.59 ± 2.2^c^
8	3,4-Dihydroxybenzoic acid	153.01933	1.860	Y = 1.71e5X	0.9986	3.4005	9.63 ± 0.64^c^	35.75 ± 1.41^a^	23.32 ± 1.51^b^	33.55 ± 2.08^a^	24.67 ± 1.53^b^
9	Epicatechin	289.07176	4.780	Y = 2.661e5X	0.9995	1.3066	1.03 ± 0.07^d^	25.39 ± 1.12^c^	34.27 ± 2.22^b^	25.22 ± 1.56^c^	40.3 ± 2.49^a^
10	Vanillic acid	167.03498	4.140	Y = 2.228e5X	0.9986	2.5679	13.01 ± 0.86^e^	29.52 ± 1.16^b^	25.12 ± 1.37^a^	20.45 ± 1.26^d^	22.77 ± 1.41^c^
11	Genistin	431.09837	5.810	Y = 2.898e4X	0.9992	3.3003	13.2 ± 0.87^c^	14.96 ± 0.59^b^	22.03 ± 1.20^a^	22.18 ± 1.37^a^	14.73 ± 0.91^bc^
12	(+)-Dihydroquercetin	303.05103	5.810	Y = 2.871e5X	0.999	1.8919	9.42 ± 0.62^c^	4.33 ± 0.17^d^	5.65 ± 0.31^d^	25.44 ± 1.57^b^	38.06 ± 1.83^a^
13	Syringic acid	197.04555	4.500	Y = 2.236e5X	0.9964	0.9271	7.36 ± 0.48^c^	13.36 ± 0.53^b^	14.29 ± 0.78^ab^	13.97 ± 0.86^ab^	14.51 ± 0.70^a^
14	4-Hydroxybenzoic acid	137.02442	3.360	Y = 3.366e5X	0.9995	1.1923	9.76 ± 0.64^c^	14.19 ± 0.56^a^	10.68 ± 0.58^c^	12.34 ± 0.76^b^	15.19 ± 0.73^a^
15	Sinapic Acid	223.0612	5.660	Y = 2.455e5X	0.9966	1.5269	1.99 ± 0.13^c^	10.54 ± 0.42^b^	14.73 ± 0.80^b^	13.63 ± 0.89^a^	13.94 ± 1.98^a^
16	Kaempferol-3-O-glucoside	447.09328	6.040	Y = 1.916e5X	0.9982	1.9708	2.84 ± 0.19^b^	6.95 ± 0.27^a^	9.65 ± 0.52^b^	5.19 ± 0.34^cd^	22.45 ± 3.19^c^
17	Vitexin	431.09837	5.580	Y = 3.08e5X	0.9991	5.9853	3.58 ± 0.17^c^	14.83 ± 0.58^a^	15.3 ± 0.83^a^	4.21 ± 0.37^c^	6.15 ± 0.87^b^
18	Salicylic acid	137.02442	5.700	Y = 4.085e5X	0.9998	3.8873	2.83 ± 0.14^d^	5.76 ± 0.23^c^	4.23 ± 0.23^cd^	11.5 ± 1.02^b^	19.04 ± 2.7^a^
19	Apigenin	269.04555	7.520	Y = 1.081e6X	0.9991	5.0684	2.37 ± 0.11^d^	22.16 ± 0.87^a^	9.02 ± 0.49^b^	2.29 ± 0.2^d^	3.4 ± 0.48^c^
20	Luteoloside	447.09328	5.720	Y = 1.499e5X	0.9994	5.9819	2.75 ± 0.13^d^	15.98 ± 1.70^a^	7.06 ± 0.26^b^	1.83 ± 0.16^d^	4.34 ± 0.49^c^
21	Vanillin	151.04007	5.120	Y = 3.766e5X	0.9984	2.0955	4.62 ± 0.22^c^	6.08 ± 0.60^b^	5.61 ± 0.21^b^	6.33 ± 0.56^b^	7.72 ± 0.87^a^
22	p-Hydroxycinnamic Acid	163.04007	5.270	Y = 4.696e5X	0.999	1.2859	4.43 ± 0.21^c^	5.07 ± 0.50^bc^	5.62 ± 0.21^b^	4.96 ± 0.44^bc^	9.76 ± 1.09^a^
23	Caffeic acid	179.03498	4.300	Y = 2.861e5X	0.9975	2.7987	2.68 ± 0.13^d^	5.04 ± 0.5^c^	6.59 ± 0.24^b^	6.08 ± 0.55^b^	8.81 ± 0.99^a^
24	Protocatechualdehyde	137.02442	3.160	Y = 3.514e5X	0.9953	0.7966	1.23 ± 0.06^d^	6.41 ± 0.63^b^	5.04 ± 0.19^c^	5.19 ± 0.79^c^	8.14 ± 0.91^a^
25	Isorhamnetin	315.05103	7.680	Y = 7.377e5X	0.9953	1.1405	0.24 ± 0.01^c^	4.02 ± 0.4^b^	3.71 ± 0.14^b^	6.92 ± 1.12^a^	7.02 ± 0.79^a^
26	Gallic acid	169.01425	0.980	Y = 1.159e5X	0.9986	2.9169	3.63 ± 0.18^c^	4.72 ± 0.46^ab^	4.98 ± 0.18^a^	2.09 ± 0.34^d^	4.34 ± 0.49^b^
27	Naringenin	271.06120	7.520	Y = 9.737e5X	0.9969	3.6767	1.22 ± 0.06^d^	3.48 ± 0.34^bc^	3.22 ± 0.12^c^	4.32 ± 0.7^b^	7.4 ± 0.83^a^
28	Syringaldehyde	181.05063	5.430	Y = 3.662e5X	0.9988	0.2795	1.9 ± 0.09^d^	5.63 ± 0.55^a^	2.84 ± 0.11^c^	3.01 ± 0.49^c^	4.14 ± 0.46^b^
29	Luteolin	285.04046	7.000	Y = 6.495e5X	0.9985	5.6980	1.2 ± 0.06^d^	6.46 ± 0.64^a^	3.79 ± 0.21^b^	2.3 ± 0.37^c^	3.65 ± 0.41^b^
30	Gossypol	517.18679	9.900	Y = 3.212e3X	0.9983	8.0612	12.18 ± 0.60^a^	ND	ND	ND	ND
31	(+)-Dihydrokaempferol	287.05611	6.390	Y = 5.659e5X	0.9959	1.5594	0.92 ± 0.05^d^	1.94 ± 0.07^c^	1.1 ± 0.06^d^	2.82 ± 0.53^b^	3.95 ± 0.44^a^
32	Naringenin Chalcone	271.0612	7.470	Y = 1.236e5X	0.9959	6.6063	2.9 ± 0.14^a^	3.03 ± 0.11^a^	1.49 ± 0.08^b^	0.97 ± 0.18^c^	1.42 ± 0.06^b^
33	Hydrocinnamic acid	149.06080	7.080	Y = 2.192e5X	0.9981	2.1695	4.88 ± 0.26^b^	0.98 ± 0.04^c^	1.24 ± 0.07^c^	1.02 ± 0.19^c^	1.01 ± 0.04^c^
34	Trans-Cinnamic acid	147.04515	7.180	Y = 1.681e5X	0.9957	2.0707	1.45 ± 0.08^a^	1.03 ± 0.04^c^	1.2 ± 0.07^b^	0.84 ± 0.03^d^	1.51 ± 0.06^a^
35	Daidzein	253.05063	6.790	Y = 8.263e5X	0.9966	1.1643	0.31 ± 0.02^e^	0.57 ± 0.02^c^	0.42 ± 0.02^d^	0.75 ± 0.03^a^	0.68 ± 0.03^b^
36	Resveratrol	227.07137	6.630	Y = 2.574e5X	0.9993	1.5947	0.12 ± 0.01^d^	0.24 ± 0.01^c^	0.34 ± 0.02^b^	0.34 ± 0.01^b^	0.37 ± 0.01^a^
37	Dihydromyricetin	319.04594	4.820	Y = 1.594e5X	0.9982	0.3397	0.42 ± 0.02^a^	ND	0.2 ± 0.01^c^	ND	0.36 ± 0.01^b^
38	Phloretin	273.07685	7.500	Y = 8.637e5X	0.9985	1.2669	ND	0.03 ± 0.00	ND	0.03 ± 0.00	0.03 ± 0.00

Note: unit: ng/100 mg. a, b, c, d and e Means with different letters within a row differ significantly (*P* < 0.05). ±: Represents the standard deviation. *n* = 3; RSD means relative standard deviations; The control sample was designated as “K”; Incorporated bread samples with Tsingtao Flower hop pellets soaked in boiling water was labeled “GK”; Utilized bread samples with Tsingtao Flower hop pellets soaked in cold water was labeled “GL”; Incorporated bread samples with Saaz hop pellets soaked in boiling water was labeled “JK”; Utilized bread samples with Saaz hop pellets soaked in cold water was labeled “JL”. Phenolic compounds were conducted in triplicate to ensure the reliability and consistency of the results.

Rutin, a flavonoid widely found in plants, is recognized for its antioxidant, anti-inflammatory, and vascular protective properties. It enhances capillary structure and helps prevent cardiovascular diseases (Singh et al., 2024). Rutin levels varied significantly among the five samples, with Tsingtao hops containing much higher amounts than those of Saaz hops (*P* < 0.05). GK had six times the JK content, while GL had eight times the JL content. Rutin, a key flavonoid in Tsingtao hops, exhibited the highest content under hot water-soaking conditions. Rutin is usually found in bound form within cell walls or intercellular spaces. Heat treatment may disrupt these structures, releasing more bound polyphenols (Farha et al., 2020). Additionally, Izham et al. (2022) reported that elevated temperatures increase water polarity and molecular mobility of rutin, thereby significantly enhancing the solubility of rutin and its overall extractable content. A similar trend was observed for trans-ferulic acid, with both hops significantly increasing their bread content and hot water soaking yielding higher levels than those of cold-water soaking. Benzoic acid, known for its strong antimicrobial properties, is commonly used as a preservative in food and cosmetics to inhibit bacterial and fungal growth (Di Domenico et al., 2020). Consistent with the trend observed for quercetin, the benzoic acid content varied by hop variety, with Tsingtao hops exhibiting the highest levels when soaked in hot water, while Saaz hops showed the opposite trend. These variations result from genetic factors, environmental conditions, and varietal differences influencing bioactive compounds in hops (Féchir et al., 2023).

The kaempferol content was higher in the cold water-treated sample than in the hot water-treated sample, independent of hop variety; however, Saaz hops contained more kaempferol. Catechin, a flavanol-type polyphenol, is a key antioxidant component in teas—especially green tea, was higher in hot water soaking than in cold, with Saaz hops having significantly more catechin than that of Tsingtao hops, establishing it as a signature compound in Saaz hops. Gossypol was detected at a concentration of 12.18 ng in bread but disappeared upon the addition of hops. Gossypol, a naturally occurring polyphenolic compound in cotton seeds and roots, exhibits antioxidant, anticancer, and antimicrobial activities. However, gossypol raises concern owing to its toxicity and reproductive inhibitory properties. Polyphenols in hops may form complexes with gossypol, causing a loss of activity or a reduction in its content. Moreover, antioxidants such as quercetin, kaempferol, and catechin in hops may inhibit further oxidation or decomposition of gossypol (Speisky et al., 2023).

### 3.4 Analysis of *E*-tongue

**Fig. 2:**
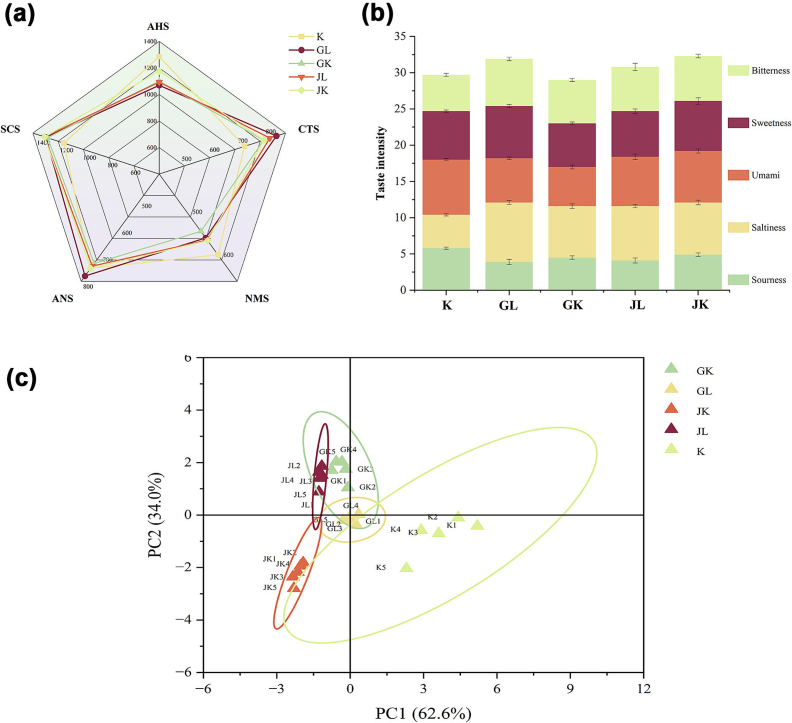
*E*-tongue profile of bread prepared using various hop varieties treated with cold and hot water soaking. Note: (a) The radar chart of the E-tongue (b) Taste intensity value by E-tongue. (c) The principal component analysis of the E-tongue.

Fig. 2(a) and (b) illustrate the taste radar chart and taste intensity, respectively. Hop addition enhanced all sensory signals related to taste perception. The AHS sensor indicated that fermentation-induced acidity decreases with hop addition, suggesting that hops inhibit lactic acid bacterial growth and excessive acid production, thereby reducing bread AHS (Kolenc et al., 2022). The CTS sensor showed that hops significantly enhanced bread CTS, particularly in the GL sample, even without salt. The GK, JL, and JK samples exhibited similar trends, although these changes were not statistically significant (*P* < 0.05). Excessive sodium intake is associated with various health risks, including cardiovascular disease, gastric cancer, obesity, osteoporosis, Meniere's disease, and kidney dysfunction (Yang et al., 2024). Hops may enhance CTS perception in the GL sample, potentially helping to reduce high sodium intake. The NMS sensor detected a reduction in NMS flavor in hop-infused bread, with similar decreasing trends in the GL, JL, and JK samples; however, the GK sample exhibited a more significant reduction. The ANS sensor showed a slight increase in ANS in the GL sample compared to that in the K sample, a minimal change in the JK sample, and a decrease in ANS in the JL and JK samples. The SCS sensor indicated that all hop-infused breads exhibited increased SCS compared to that of the control, possibly due to the presence of bitter acids, monoterpenes, and sesquiterpenes (Dietz et al., 2020). This phenomenon aligns with that of previous research (Nionelli et al., 2018).

Fig. 2(c) depicts the flavor differences among samples. Non-overlapping sample distances demonstrated different flavor profiles. The flavor of the hops-infused bread differed significantly from that of the K sample. While the flavors of the hop bread sample were similar, they remained distinct. The JL and GK samples exhibited similarity, while the GL sample was centrally positioned, differing from the others. The JK was equidistant from the other hop bread samples in the third quadrant. Abdus-Salaam et al. (2024) reported that the soaking method used for extracting papaya leaf compounds had a significant impact on bread flavor, leading to improved sensory qualities and overall consumer acceptability. These findings suggest that the soaking method significantly influence the flavor profile of the final hop bread.

### 3.5 Analysis of amino acids

FAA is crucial for the flavor and color of food, acting as precursors to VOCs (Liu et al., 2022). In addition to their role in the essential Maillard reaction, protein, or peptide hydrolysis during bread baking leads to amino acid condensation, the Amadori–Heyns rearrangement, and enolization, all vital for developing bread flavor (Yang et al., 2022). Different amino acids exhibit distinct taste characteristics, including SCS, NMS, and ANS; thus, their variety and concentration significantly affect bread flavor.

Fifteen FAA were identified (Table 3). The total free amino acids in the K sample amounted to 48.50 μg/100 g, while reductions in the GK, GL, JK, and JL samples were by factors of 4.78, 3.03, 2.23, and 3.23, respectively. The amino acid content of all samples decreased significantly after the addition of hops (*P* < 0.05), except for lysine and threonine. Seven of the 15 amino acids, including histidine, leucine, phenylalanine, valine, tyrosine, isoleucine, and lysine, were identified as bitter. This indicates that bitter compounds significantly contribute to the taste of bread. Moderate SCS enhances overall flavor perception, with these bitter compounds being responsible for the distinctive taste of hop bread (Chu et al., 2024). Sweet amino acids include alanine, proline, arginine, serine, glycine, and threonine, while glutamic and aspartic acids affect NMS flavor. Despite the limited number of NMS compounds, glutamic acid remains the most abundant, serving as a crucial determinant of the final flavor of the bread.

**Table 3:** Amino acid content of fermented meat under different reheating methods.

Amino acids	Taste characteristics	K	GL	GK	JL	JK
Glutamic acid (Glu)	Umami (+)	10.47 ± 1.51^a^	3.05 ± 0.12^c^	4.54 ± 0.78^bc^	4.73 ± 0.87^bc^	6.43 ± 1.12^b^
Alanine (Ala)	Sweet (+)	4.06 ± 0.13^a^	1.26 ± 0.24^d^	1.90 ± 0.12^c^	2.63 ± 0.67^b^	2.61 ± 0.34^b^
Aspartic acid (Asp)	Umami (+)	3.51 ± 0.27^a^	1.57 ± 0.11^c^	1.46 ± 0.41^cd^	1.29 ± 0.11^d^	2.67 ± 0.21^b^
Histidine (His)⁎	Bitter (−)	3.60 ± 1.01^a^	1.07 ± 0.08^d^	1.52 ± 0.34^c^	1.95 ± 0.86^b^	1.97 ± 0.14^b^
Leucine (Leu)⁎	Bitter (−)	6.53 ± 2.10^a^	0.10 ± 0.01^b^	0.83 ± 0.04^b^	0.08 ± 0.01^b^	0.55 ± 0.04^b^
Proline (Pro)	Sweet (+)	4.10 ± 0.24^a^	0.42 ± 0.22^b^	0.80 ± 0.31^b^	0.96 ± 0.41^b^	0.75 ± 0.07^b^
Phenylalanine (Phe)⁎	Bitter (−)	4.47 ± 0.57^a^	0.26 ± 0.06^c^	0.52 ± 0.12^b^	0.29 ± 0.07^c^	0.65 ± 0.13^b^
Arginine (Arg)	Sweet (+)	1.96 ± 0.18^a^	0.68 ± 0.11^d^	1.57 ± 0.11^b^	0.81 ± 0.16^cd^	1.04 ± 0.22^c^
Valine (Val)⁎	Bitter (−)	2.61 ± 0.12^a^	0.38 ± 0.06^d^	0.52 ± 0.03^cd^	0.71 ± 0.18^bc^	0.84 ± 0.12^b^
Tyrosine (Tyr)	Bitter (−)	1.80 ± 0.15^a^	0.35 ± 0.08^d^	0.87 ± 0.09^b^	0.41 ± 0.22^cd^	0.58 ± 0.14^c^
Isoleucine (Ile)⁎	Bitter (−)	2.07 ± 0.20^a^	0.13 ± 0.02^c^	0.46 ± 0.02^b^	0.12 ± 0.01^c^	0.27 ± 0.06^c^
Lysine (Lys)⁎	Bitter (−)	0.82 ± 0.07^b^	0.23 ± 0.01^c^	0.23 ± 0.01^c^	0.23 ± 0.03^c^	1.21 ± 0.23^a^
Serine (Ser)	Sweet (+)	1.22 ± 0.11^a^	0.27 ± 0.03^c^	0.26 ± 0.02^c^	0.03 ± 0.01^d^	0.79 ± 0.17^b^
Glycine (Gly)	Sweet (+)	0.64 ± 0.06^a^	0.24 ± 0.10^c^	0.35 ± 0.01^bc^	0.59 ± 0.15^a^	0.50 ± 0.16^ab^
Threonine (Thr)⁎	Sweet (+)	0.63 ± 0.09^b^	0.15 ± 0.05^c^	0.18 ± 0.03^a^	0.20 ± 0.04^c^	0.87 ± 0.09^c^
Total Free Amino Acid Content (TFAA)		48.50 ± 6.81^a^	10.15 ± 1.30^d^	16.01 ± 2.44^c^	21.73 ± 3.24^b^	15.02 ± 3.80^c^

⁎ represents essential amino acids (EAA); n = 3; unit: μg/100 g; “(+)” indicates a pleasant taste; “(−)” indicates an unpleasant taste. Note: a, b, c, d and e Means with different letters within a row differ significantly (*P* < 0.05). ±: Represents the standard deviation. *n* = 3; The control sample was designated as “K”; Tsingtao Flower hop pellets soaked in boiling water was labeled “GK”; Tsingtao Flower hop pellets soaked in cold water was labeled “GL”; Saaz hop pellets soaked in boiling water was labeled “JK”; Saaz hop pellets soaked in cold water was labeled “JL”. Free amino acids were conducted in triplicate to ensure the reliability and consistency of the results.

Furthermore, an observation indicated that soaking methods and hop varieties significantly affected amino acid content. Cold-water-soaked hops caused the greatest reduction in amino acid content in bread, followed by hot-water-soaked hops. Tsingtao hops consumed more amino acids than those of Saaz hops, regardless of soaking temperature. This suggests that compounds in Tsingtao hops may undergo complex chemical reactions with starch and amino acids during fermentation and baking, including 1,2-enolation, 2,3-enolation, Strecker degradation, and Amadori and Heyns rearrangements, leading to the formation of a wide variety of volatile compounds. In particular, amino acids involved in Strecker degradation contribute to the generation of branched volatiles such as esters, alcohols, and ketones, which are recognized as the primary aroma-active compounds in bread (Yang et al., 2022).

### 3.6 Analysis of *E*-nose

**Fig. 3:**
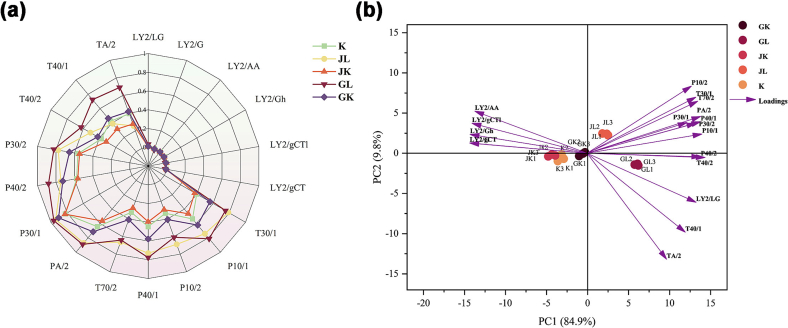
E-nose profile of bread prepared using various hop varieties treated with cold and hot water soaking. Note: (a) The radar chart of the E-nose, (b) The principal component analysis of the E-nose.

Fig. 3(a) presents the radar chart for hop bread. All sensors, except LY2, responded to bread aroma, with the highest intensities in P30/1, P40/2, and PA/2, indicating a prevalence of aldehydes and alcohols. GL exhibited significantly stronger signals for P40/2 and PA/2 than those of other samples. The response intensities for P30/1, P40/2, and PA/2 varied among the samples. Hop enhanced the aroma in nearly all samples compared to that in the K sample, with GL and JL showing significant improvements. GL showed a richer aroma intensity than that of JL, followed by GK and JK, indicating that bread made with cold water-soaked Tsingtao hops exhibited significant aroma.

PCA was employed to visually distinguish the samples, simplify data complexity, and eliminate redundant variables (Ping et al., 2024). The model accounted for 95.7 % of the total variance (PC1 = 84.9 %, PC2 = 9.8 %), enabling clear sample distinction (Fig. 3b). The GL and JL samples were positioned on the right side of the axis but exhibited significant aromatic differences. GL located in the fourth quadrant, was separated from the other samples, indicating significant aromatic variance. JL, situated in the first quadrant, was positioned closer to the remaining three samples. JK, K, and GK clustered together, with some overlap between JK and K, suggesting similar aromatic properties, while GK was positioned farther from K, indicating greater distinction. These results suggest that hop variety and extraction temperature jointly influence the aroma profile of sourdough bread. Notably, GL led to a distinct volatile composition, potentially due to selective solubilization of specific aroma-active compounds under mild conditions.

The *E*-nose effectively differentiates products based on aroma; however, it cannot identify the specific compounds responsible for these variations. Therefore, HS-GC-IMS was employed to identify the key aroma compounds in the bread samples.

### 3.7 Analysis of HS-GC-IMS

**Fig. 4:**
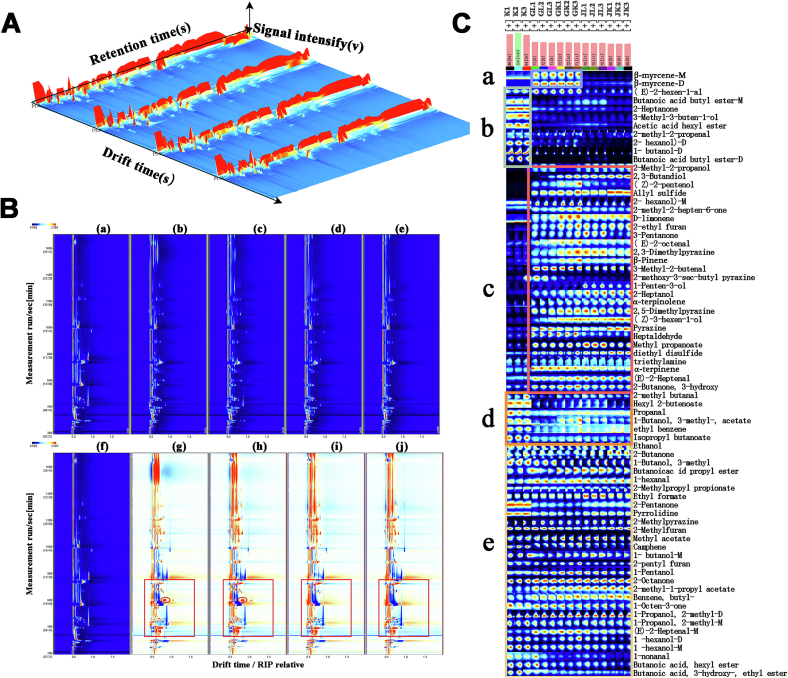
Analysis of HS-GC-IMS after different reheating methods. Note: The control sample was designated as “K”; Tsingtao Flower hop pellets soaked in boiling water was labeled “GK”; Tsingtao Flower hop pellets soaked in cold water was labeled “GL”; Saaz hop pellets soaked in boiling water was labeled “JK”; Saaz hop pellets soaked in cold water was labeled “JL”. (A). The three-dimensional spectrum of volatile compounds in K, GK, GL, JL, JK (B). Composition spectrum (top view) and difference spectrum of volatile compounds. (a) 2D qualitative chromatogram of K samples (b) 2D qualitative chromatogram of GL samples (c) 2D qualitative chromatogram of GK samples (d) 2D qualitative chromatogram of JL samples (e) 2D qualitative chromatogram of JK samples (f) Difference chromatogram of K samples (g) Difference chromatogram of GL samples (h) Difference chromatogram of GK samples (i) Difference chromatogram of JL samples (j) Difference chromatogram of JK samples. (C). Fingerprint spectrum of samples gallery plot.

Table 4 presents the distinct identification of 71 VOCs in hop bread, categorized as follows: seven acids, eighteen alcohols, thirteen esters, eleven aldehydes, eight ketones, seven alkenes, five pyrazines, three furans, two benzenes, two amines, and two sulfides. Fig. 4(A) presents a 3D topographic map illustrating the volatile flavor profile of hop bread. The X-axis represents ion migration time (ms), Y-axis denotes retention time (s), and *Z*-axis represents the intensity value of the compounds (v). The red vertical line on the left marks the reactive ion peak, with each dot representing a distinct compound. Color depth indicates peak intensity, with blue signifying lower intensity and red indicating higher intensity. Although the 3D map provides a comprehensive top-down overview of flavor changes across hop bread samples, it lacks resolution in distinguishing variations within specific migration time ranges. To address this limitation, the spectra of hop bread (f) served as a reference for comparing the VOC variations among the samples. In Fig. 4 (B), the white background indicates VOC levels, while red and blue denote higher and lower values, respectively, relative to the reference. Most compound signals are observed within a retention time range of 300–1400 s. At a migration time of approximately 600 s, significant enhancements in compound response signals were observed in the GL and GK samples, while the JK and JL samples exhibited no response signals. These differences may contribute to the observed flavor variations.

**Table 4:** Peak intensity of K, JL, JK, GL, GK samples.

Count	Classification	Compounds	CAS	Formula	Structural type	Molecular weight	Retention time	Retention indexes	Drift time	Peak Intensity				
										K	GL	GK	JL	JK
1	Alcohols	2,3-Butanediol	513–85-9	C4H10O2		90.1	1731.119	1532.7	1.36284	1207.69 ± 819.96^c^	7739.13 ± 6263.57^b^	24,328.38 ± 3533.21^a^	25,856.61 ± 460.50^a^	28,308.35 ± 1523.60^a^
2		butanol-D	71–36-3	C4H10O		74.1	578.825	1146.3	1.38902	40,991.45 ± 665.83^a^	7403.11 ± 3321.48^b^	7472.77 ± 609.19^b^	8403.07 ± 444.49^b^	3554.28 ± 195.23^c^
3		isobutanol-D	78–83-1	C4H10O		74.1	460.415	1092.1	1.37831	9557.24 ± 615.62^b^	11,934.93 ± 362.43^a^	12,343.79 ± 505.19^a^	12,203.82 ± 89.19^a^	12,097.98 ± 141.60^a^
4		(Z)-3-hexen-1-ol	928–96-1	C6H12O		100.2	1300.639	1389.1	1.21863	2533.11 ± 378.49^c^	6169.11 ± 1995.44^b^	9782.56 ± 645.70^a^	9792.73 ± 317.95^a^	10,770.14 ± 57.63^a^
5		2-hexanol-M	626–93-7	C6H14O		102.2	697.11	1196.3	1.26755	4868.70 ± 119.33^a^	3914.84 ± 174.71^b^	4103.65 ± 126.68^b^	2912.12 ± 147.99^c^	2886.61 ± 59.02^c^
6		1-hexanol-M	111–27-3	C6H14O		102.2	1220.481	1362.4	1.32916	4434.69 ± 74.08^a^	3292.27 ± 223.68^d^	4032.62 ± 140.27^b^	3746.50 ± 86.34^c^	2871.38 ± 86.89^e^
7		pentanol	71–41-0	C5H12O		88.1	896.909	1257.7	1.25729	2868.80 ± 55.07^b^	2912.06 ± 130.90^b^	3065.61 ± 46.27^a^	2679.07 ± 70.14^c^	2308.93 ± 28.81^d^
8		1-hexanol-D	111–27-3	C6H14O		102.2	1216.987	1361.2	1.64447	1574.45 ± 36.65^a^	891.40 ± 124.16^b^	1439.59 ± 292.55^a^	1120.71 ± 90.90^b^	601.59 ± 34.33^c^
9		isobutanol	78–83-1	C4H10O		74.1	472.55	1099.7	1.1767	1255.06 ± 89.72^a^	1026.37 ± 15.22^c^	1018.53 ± 23.20^c^	1159.66 ± 21.63^b^	1090.83 ± 15.64^bc^
10		3-methyl-1-butanol-M	123–51-3	C5H12O		88.1	749.762	1212.4	1.25389	1874.30 ± 99.00^a^	449.37 ± 32.89^d^	984.39 ± 126.81^c^	1287.35 ± 142.48^b^	622.11 ± 36.85^d^
11		ethanol	64–17-5	C2H6O		46.1	285.549	895.7	1.12408	1092.81 ± 57.09^a^	665.66 ± 39.11^c^	542.99 ± 36.74^d^	763.90 ± 11.78^b^	792.40 ± 27.32^b^
12		isoprenol	763–32-6	C5H10O		86.1	882.564	1253.3	1.17991	1669.89 ± 88.24^a^	426.15 ± 63.08^b^	237.21 ± 10.08^d^	307.75 ± 19.43^cd^	373.42 ± 4.32^bc^
13		butanol	71–36-3	C4H10O		74.1	556.155	1136.3	1.18153	543.65 ± 57.17^b^	489.42 ± 88.07^b^	570.35 ± 24.63^b^	680.47 ± 9.48^a^	560.53 ± 17.34^b^
14		2-heptanol	543–49-7	C7H16O		116.2	1155.869	1340.9	1.38276	247.21 ± 33.23^c^	527.57 ± 41.79^b^	573.26 ± 27.66^b^	710.77 ± 31.60^a^	712.99 ± 34.18^a^
15		1-penten-3-ol	616–25-1	C5H10O		86.1	599.586	1155.4	0.94051	331.51 ± 15.77^d^	444.96 ± 15.49^c^	470.54 ± 32.39^c^	803.29 ± 18.09^a^	682.38 ± 10.91^b^
16		2-hexanol-D	626–93-7	C6H14O		102.2	669.249	1185.9	1.55666	1057.96 ± 20.80^a^	342.59 ± 47.68^c^	465.60 ± 60.95^b^	153.12 ± 14.95^d^	129.31 ± 8.58^d^
17		cis-2-Penten-1-ol	1576-95-0	C5H10O		86.1	1120.737	1329.1	1.44833	66.04 ± 7.21^d^	483.34 ± 51.78^b^	744.60 ± 143.75^a^	405.53 ± 65.83^bc^	280.13 ± 22.41^c^
18		tert-butanol	75–65-0	C4H10O		74.1	295.562	913.1	1.12549	81.75 ± 60.85^b^	327.33 ± 56.38^a^	141.96 ± 44.09^b^	345.01 ± 22.54^a^	398.41 ± 43.73^a^
19	Esters	ethyl 3-hydroxybutyrate	5405-41-4	C6H12O3		132.2	1651.072	1506	1.16347	10,833.95 ± 1409.78^a^	9997.47 ± 1416.06^a^	7624.95 ± 198.83^b^	7065.89 ± 293.09^b^	6416.62 ± 123.27^b^
20		butyl butanoate-D	109–21-7	C8H16O2		144.2	772.723	1219.5	1.81611	23,722.89 ± 675.34^a^	1140.94 ± 276.27^c^	1197.00 ± 77.75^c^	2988.05 ± 422.03^b^	832.64 ± 56.28^c^
21		methyl propanoate	554–12-1	C4H8O2		88.1	283.532	892.2	1.33624	1459.75 ± 79.99^c^	4130.82 ± 309.67^b^	3974.32 ± 853.79^b^	6282.85 ± 194.81^a^	3585.85 ± 290.68^b^
22		lsobutyl acetate	110–19-0	C6H12O2		116.2	338.007	987	1.22173	1353.88 ± 94.46^c^	1967.90 ± 44.77^b^	2045.68 ± 63.59^b^	2153.97 ± 12.77^a^	2180.02 ± 40.72^a^
23		hexyl butyrate	2639-63-6	C10H20O2		172.3	1322.951	1396.6	1.48743	2445.67 ± 83.77^b^	2122.43 ± 598.12^bc^	1585.91 ± 112.78^b^	1769.15 ± 138.79^cd^	1114.59 ± 113.43^d^
24		lsobutyl propionate	540–42-1	C7H14O2		130.2	452.378	1085.5	1.2752	1671.59 ± 146.79^a^	1375.17 ± 59.77^b^	1223.94 ± 38.64^c^	1452.21 ± 42.33^b^	1402.10 ± 26.38^b^
25		ethyl formate	109–94-4	C3H6O2		74.1	251.093	835.8	1.06989	1333.02 ± 96.48^b^	1396.22 ± 12.40^b^	1361.96 ± 37.13^b^	1659.59 ± 72.38^a^	1368.24 ± 33.59^b^
26		hexyl crotonate	19,089–92-0	C10H18O2		170.3	787.314	1224	1.44835	1379.57 ± 56.98^a^	566.02 ± 65.37^c^	825.57 ± 89.94^b^	798.59 ± 87.25^b^	552.27 ± 37.12^c^
27		lsopropyl butanoate	638–11-9	C7H14O2		130.2	392.234	1036.2	1.26274	1012.39 ± 23.66^a^	569.49 ± 15.21^c^	648.45 ± 48.68^b^	488.26 ± 16.98^d^	430.93 ± 6.66^e^
28		propyl butyrate	105–66-8	C7H14O2		130.2	555.288	1135.9	1.26359	354.45 ± 15.40^c^	497.11 ± 16.99^b^	599.04 ± 20.06^ab^	663.06 ± 9.30^a^	532.99 ± 9.99^ab^
29		hexyl acetate-M	142–92-7	C8H16O2		144.2	934.016	1269.1	1.39942	106.89 ± 17.71^e^	2279.42 ± 37.56^a^	1219.25 ± 36.84^d^	1938.56 ± 37.89^b^	1479.74 ± 29.60^c^
30		methyl acetate	79–20-9	C3H6O2		74.1	254.473	841.6	1.19724	337.04 ± 15.62^a^	312.52 ± 19.34^ab^	265.32 ± 37.42^c^	291.78 ± 14.61^bc^	251.49 ± 20.87^c^
31		lsoamyl acetate	123–92-2	C7H14O2		130.2	517.474	1119.4	1.30864	229.52 ± 18.50^a^	125.65 ± 13.48^c^	156.84 ± 16.38^b^	152.92 ± 5.01^b^	117.39 ± 14.07^c^
32	Aldehyde	(E)-2-heptenal-M	18,829–55-5	C7H12O		112.2	1105.608	1324.1	1.25566	1241.46 ± 36.18^d^	2261.13 ± 128.54^a^	2243.85 ± 142.68^a^	1941.18 ± 118.74^b^	1673.77 ± 126.26^c^
33		3-methyl-2-butenal	107–86-8	C5H8O		84.1	718.603	1202.9	1.09312	426.36 ± 11.73^d^	2119.07 ± 100.30^a^	1846.92 ± 93.19^b^	1377.05 ± 109.73^c^	1439.25 ± 87.55^c^
34		heptanal	111–71-7	C7H14O		114.2	669.78	1186.1	1.34879	1837.60 ± 46.55^b^	832.24 ± 36.21^e^	2051.00 ± 9.87^a^	1237.05 ± 40.72^c^	1116.96 ± 52.73^d^
35		methacrolein	78–85-3	C4H6O		70.1	283.666	892.4	1.22183	2025.69 ± 109.73^a^	1160.80 ± 51.75^b^	1277.19 ± 285.09^b^	726.33 ± 20.37^c^	1214.29 ± 29.22^b^
36		(E)-2-heptenal	18,829–55-5	C7H12O		112.2	1153.512	1340.1	1.25852	944.40 ± 20.15^c^	1234.13 ± 91.22^ab^	1263.78 ± 51.00^a^	1190.20 ± 65.65^ab^	1145.75 ± 34.31^b^
37		(E)-2-octenal	2548-87-0	C8H14O		126.2	1446.768	1437.9	1.33381	690.79 ± 45.66^d^	1069.86 ± 118.42^c^	1468.38 ± 36.33^a^	1242.88 ± 11.85^b^	1168.84 ± 18.87^b^
38		(E)-2-hexenal	6728-26-3	C6H10O		98.1	770.684	1218.9	1.18394	1240.70 ± 110.38^b^	1424.44 ± 138.11^a^	1274.46 ± 16.64^b^	675.58 ± 23.49^d^	828.70 ± 30.50^c^
39		hexanal	66–25-1	C6H12O		100.2	472.55	1099.7	1.25715	682.39 ± 18.71^b^	841.15 ± 67.51^a^	852.00 ± 98.91^a^	878.66 ± 54.54^a^	864.43 ± 52.77^a^
40		propanal	123–38-6	C3H6O		58.1	220.439	782.4	1.04051	954.02 ± 103.62^a^	739.47 ± 39.29^b^	725.27 ± 20.64^b^	769.11 ± 50.98^b^	744.62 ± 35.11^b^
41		2-methyl butanal	96–17-3	C5H10O		86.1	297.318	916.2	1.40023	420.69 ± 40.31^e^	895.71 ± 365.45^a^	677.96 ± 131.74^c^	805.08 ± 105.42^b^	637.74 ± 79.48^d^
42		nonanal	124–19-6	C9H18O		142.2	1323.404	1396.7	1.93793	449.07 ± 57.37^a^	397.16 ± 119.14^ab^	294.18 ± 9.81^b^	371.44 ± 31.57^ab^	283.44 ± 14.70^b^
43	Ketones	2-octanone	111–13-7	C8H16O		128.2	1011.475	1292.9	1.32985	25,850.06 ± 371.66^d^	34,663.67 ± 446.55^a^	31,311.53 ± 628.33^c^	31,944.42 ± 142.62^c^	32,266.85 ± 222.73^b^
44		acetoin	513–86-0	C4H8O2		88.1	891.527	1256	1.3344	1580.22 ± 64.24^c^	2905.84 ± 118.00^b^	3644.73 ± 253.30^a^	3087.36 ± 181.71^b^	2970.06 ± 41.72^b^
45		2-pentanone	107–87-9	C5H10O		86.1	366.688	1015.3	1.13593	3124.21 ± 131.55^a^	1439.65 ± 42.72^c^	1146.32 ± 27.17^d^	1642.81 ± 49.29^b^	1675.16 ± 44.50^b^
46		sulcatone	110–93-0	C8H14O		126.2	1151.142	1339.3	1.17868	1133.23 ± 104.97^bc^	1314.87 ± 65.74^b^	1563.54 ± 157.42^a^	1026.28 ± 101.15^cd^	858.67 ± 47.67^d^
47		2-butanone	78–93-3	C4H8O		72.1	286.678	897.7	1.0493	928.43 ± 169.32^b^	991.67 ± 65.03^b^	872.06 ± 125.13^b^	907.54 ± 42.97^b^	1271.01 ± 30.12^a^
48		2-heptanone	110–43-0	C7H14O		114.2	655.466	1179.8	1.26762	1735.59 ± 49.97^a^	782.14 ± 96.21^b^	652.59 ± 7.07^c^	593.56 ± 19.00^c^	407.41 ± 10.29^d^
49		3-pentanone	96–22-0	C5H10O		86.1	336.869	985	1.36254	192.60 ± 13.67^d^	725.63 ± 63.53^b^	816.28 ± 159.26^ab^	979.17 ± 93.52^a^	483.29 ± 47.30^c^
50		1-octen-3-one	4312-99-6	C8H14O		126.2	1050.236	1305.6	1.28065	382.77 ± 44.16^e^	697.91 ± 30.78^a^	529.76 ± 39.23^c^	612.31 ± 5.45^b^	497.97 ± 17.10^d^
51	Alkenes	α-pinene	80–56-8	C10H16		136.2	392.846	1036.7	1.20728	886.37 ± 85.61^e^	3102.28 ± 24.56^a^	2924.67 ± 81.93^b^	2651.26 ± 5.10^c^	2331.46 ± 39.80^d^
52		camphene	79–92-5	C10H16		136.2	402.642	1044.7	1.20005	2293.46 ± 26.70^a^	1855.17 ± 145.53^b^	2452.14 ± 177.46^a^	1851.44 ± 180.50^b^	1396.41 ± 79.31^c^
53		β-myrcene-M	123–35-3	C10H16		136.2	600.477	1155.7	1.21444	358.44 ± 14.09^e^	2569.47 ± 95.07^b^	2683.13 ± 58.37^a^	927.88 ± 35.91^d^	1032.39 ± 12.33^c^
54		terpinolene	586–62-9	C10H16		136.2	987.477	1285.5	1.20717	338.75 ± 67.02^d^	892.00 ± 123.59^c^	1112.53 ± 17.87^b^	1118.39 ± 24.48^b^	1267.45 ± 72.21^a^
55		β-myrcene-D	123–35-3	C10H16		136.2	600.295	1155.7	1.28925	503.84 ± 12.95^c^	1259.47 ± 16.18^b^	1199.74 ± 14.25^a^	228.24 ± 16.05^c^	168.11 ± 9.94^d^
56		β-pinene	127–91-3	C10H16		136.2	541.411	1129.9	1.22175	237.12 ± 42.23^e^	650.44 ± 27.48^c^	434.23 ± 68.50^d^	681.13 ± 12.10^b^	728.12 ± 4.37^a^
57		limonene	138–86-3	C10H16		136.2	686.775	1193.1	1.20993	156.38 ± 7.76^e^	996.34 ± 63.54^a^	690.56 ± 18.18^d^	715.90 ± 4.02^c^	851.55 ± 5.30^b^
58	Pyrazines	2-methoxy-3-s-butyl pyrazine	24,168–70-5	C9H14N2O		166.2	1644.984	1504	1.27203	3229.32 ± 3008.06^a^	5060.03 ± 1627.52^a^	1835.87 ± 328.33^b^	1284.35 ± 116.25^b^	906.21 ± 124.23^b^
59		pyrazine	290–37-9	C4H4N2		80.1	755.505	1214.2	1.04344	807.99 ± 23.32^d^	2640.91 ± 198.49^b^	2299.07 ± 77.95^c^	2194.28 ± 100.63^c^	3038.75 ± 76.40^a^
60		2,5-dimethylpyrazine	123–32-0	C6H8N2		108.1	1107.454	1324.7	1.50524	312.63 ± 17.56^c^	1344.06 ± 103.29^b^	1618.19 ± 42.58^a^	1669.70 ± 148.34^a^	1391.21 ± 55.97^b^
61		2-methylpyrazine	109–08-0	C5H6N2		94.1	985.325	1284.8	1.07471	606.67 ± 110.57^b^	898.28 ± 58.09^a^	923.23 ± 9.52^a^	932.37 ± 17.63^a^	926.66 ± 9.74^a^
62		2,3-dimethylpyrazine-D	5910-89-4	C6H8N2		108.1	1214.827	1360.5	1.47507	352.45 ± 8.62^d^	567.89 ± 88.80^c^	944.46 ± 121.32^ab^	1022.05 ± 10.20^a^	876.38 ± 24.80^b^
63	Furans	2-methylfuran	534–22-5	C5H6O		82.1	269.91	868.5	0.9939	6306.38 ± 238.12^b^	7195.67 ± 159.91^a^	6046.11 ± 342.70^b^	6444.35 ± 442.88^b^	7131.22 ± 55.91^a^
64		2-amylfuran	3777-69-3	C9H14O		138.2	812.497	1231.7	1.25389	3640.27 ± 68.68^a^	2948.70 ± 190.95^b^	3098.90 ± 258.88^b^	2398.95 ± 29.79^c^	2471.60 ± 39.92^c^
65		2-ethyl furan	3208-16-0	C6H8O		96.1	325.942	966	1.31395	33.97 ± 9.42^d^	101.04 ± 2.72^c^	132.54 ± 5.58^a^	112.47 ± 7.59^bc^	123.20 ± 6.03^ab^
66	Benzenes	butylbenzene	104–51-8	C10H14		134.2	1032.932	1299.9	1.20686	2461.44 ± 59.80^c^	2804.85 ± 46.83^bc^	3065.36 ± 377.86^ab^	3157.38 ± 65.02^ab^	3424.46 ± 301.82^a^
67		ethyl benzene	100–41-4	C8H10		106.2	536.208	1127.6	1.07694	301.42 ± 27.53^a^	140.02 ± 32.03^b^	140.00 ± 16.04^b^	115.25 ± 8.66^b^	107.93 ± 2.94^b^
68	Sulfurs	diethyl disulfide	110–81-6	C4H10S2		122.2	701.894	1197.7	1.13361	2811.16 ± 318.44^d^	5038.52 ± 245.57^bc^	4582.26 ± 510.49^c^	5544.23 ± 255.29^b^	6315.70 ± 270.41^b^
69		diallyl sulfide	592–88-1	C6H10S		114.2	581.951	1147.6	1.13738	208.67 ± 13.84^d^	2874.51 ± 430.53^c^	3405.67 ± 212.29^b^	3170.20 ± 107.47^bc^	3881.54 ± 67.81^a^
70	Amines	pyrrolidine	123–75-1	C4H9N		71.1	380.654	1026.7	1.04292	3456.08 ± 81.38^a^	2587.31 ± 73.26^b^	2125.06 ± 155.58^c^	2738.6 ± 112.47^b^	2627.13 ± 57.44^b^
71		triethylamine	121–44-8	C6H15N		101.2	232.206	802.9	1.10174	900.90 ± 73.84^cd^	788.87 ± 72.61^d^	1013.58 ± 46.01^b^	1192.05 ± 81.25^a^	1014.17 ± 30.83^b^

Note: a, b, c, d and e Means with different letters within a row differ significantly (P < 0.05). ±: Represents the standard deviation. n = 3; The control sample was designated as “K”; Incorporated bread samples with Tsingtao Flower hop pellets soaked in boiling water was labeled “GK”; Utilized bread samples with Tsingtao Flower hop pellets soaked in cold water was labeled “GL”; Incorporated bread samples with Saaz hop pellets soaked in boiling water was labeled “JK”; Utilized bread samples with Saaz hop pellets soaked in cold water was labeled “JL”. This experiment was conducted in triplicate to ensure the reliability and consistency of the results.

To identify changes in VOCs in hop bread and analyze the differences among various soaking methods, a fingerprint map was generated using the Gallery plot plugin (Fig. 4C). The fingerprint pattern was segmented into the following five zones: a (purple dashed frame), b (green), c (pink), d (orange), and e (yellow). In zone (a), β-myrcene-M and β-myrcene-D, the most abundant compounds in Tsingtao hops, served as distinguishing markers between Tsingtao and Saaz hops. As the principal component of hop essential oil, β-myrcene imparts green and citrus flavors to the bread and exhibits significant antimicrobial properties (Połeć et al., 2020). Zone (b) contained nine VOCs, including three alcohols, three esters, two aldehydes, and one ketone, which characterized the control bread but decreased or disappeared with additional hop. Butanoic acid butyl ester D, which showed minimal response, was a typical compound in the K sample. In zone (c), most VOCs—except for 2-hexanol-M, diethyl disulfide, triethylamine, α-terpinene, (E)-2-heptenal, 2-butanone, and 3-hydroxymethyl-2-hepten-6-one—were predominantly detected in hop bread, indicating a significant enhancement of the flavor profile due to hops. Zone (d) featured six VOCs, including two aldehydes and two esters, with the highest concentration observed in K sample. Zone (e) included 28 VOCs, primarily aldehydes, esters, and ketones, which were present in all samples with no significant differences observed.

### 3.8 Analysis of multivariate statistical analysis

#### 3.8.1 Analysis of key compounds

In addition to content relevance, the threshold values of VOCs must be considered as they determine their actual contribution to the overall aroma profile. The ROAV was employed to evaluate the contribution of VOCs to the total aroma, providing insights into the effects of key flavor compounds. For bread samples, a ROAV exceeding 100 indicates a significant contribution to flavor. VOCs with ROAV >1 were regarded as significantly affecting flavor, while those with ROAV between 0.1 and 1 have a moderate influence. Acetoin stands out due to its high concentration in hop bread and low odor threshold (0.5 μg/kg). Therefore, acetoin was selected as the ROAV benchmark at 100 (Table 5).

**Table 5:** The relative odor activity value of volatile organic compounds.

Classification	Chemical compounds	Flavor description^A^	Odor Threshold value(μg/kg) ^B^	ROAV				
				K	GL	GK	JL	JK
Ketones	acetoin	Butter, floral	0.5	100.00 ± 0.00	100.00 ± 0.00	100.00 ± 0.00	100.00 ± 0.00	100.00 ± 0.00
	1-octen-3-one	Potato-like, sweet	0.03	18.85 ± 2.80^e^	73.60 ± 0.45^a^	57.66 ± 1.67^c^	69.59 ± 0.87^b^	47.65 ± 0.88^d^
	3-pentanone	Fruity	2	3.06 ± 0.33^d^	6.24 ± 0.40^b^	5.57 ± 0.72^b^	7.92 ± 0.32^a^	4.07 ± 0.38^c^
	2-octanone	Floral	50.3	9.12 ± 0.77^e^	16.27 ± 0.45^a^	11.87 ± 0.52^b^	10.66 ± 0.62^d^	11.13 ± 0.11^c^
	sulcatone	Lemon, caramel, pepper	50	0.72 ± 0.09^a^	0.45 ± 0.01^b^	0.43 ± 0.01^b^	0.33 ± 0.01^c^	0.29 ± 0.01^c^
	2-heptanone	Pear, apple	140	0.39 ± 0.01	0.10 ± 0.01	0.06 ± 0.00	0.07 ± 0.00	0.05 ± 0.00
	2-butanone	Floral, fruity	50,000	ND	ND	ND	ND	ND
	2-pentanone	Milk, floral, fruity, wine	70,000	ND	ND	ND	ND	ND
Furans	2-methylfuran	Vanilla	2.3	86.89 ± 5.78^a^	53.87 ± 1.50^b^	36.27 ± 4.39^d^	45.59 ± 5.55^c^	52.2 ± 0.46^bc^
	2-pentylfuran	meaty, caramel	6	19.23 ± 1.09^a^	8.45 ± 0.21^b^	7.08 ± 0.29^c^	6.49 ± 0.31^c^	6.93 ± 0.02^c^
	2-ethyl furan	Butter, burnt, coffee	2.3	0.47 ± 0.15^c^	0.76 ± 0.01^b^	0.79 ± 0.04^b^	0.79 ± 0.02^b^	0.90 ± 0.04^b^
Sulfurs	diethyl disulfide	Cabbage, roasted onion, alliaceous, garlic	2	44.39 ± 3.20^b^	43.39 ± 2.75^b^	31.37 ± 1.36^c^	44.92 ± 0.72^b^	53.17 ± 2.60^a^
	diallyl sulfide	Garlic	32.5	0.20 ± 0.02^c^	1.53 ± 0.28^b^	1.44 ± 0.04^b^	1.58 ± 0.05^b^	2.01 ± 0.03^a^
	ethyl benzene	Sweet	8.8	1.08 ± 0.06^b^	0.27 ± 0.05^c^	0.22 ± 0.01^c^	0.21 ± 0.00^c^	0.21 ± 0.01^c^
Aldehydes	2-methyl butanal	Malty, nuts, coffee, caramel	0.8	9.04 ± 0.49^e^	35.78 ± 15.92^a^	11.59 ± 1.80^c^	19.09 ± 1.08^b^	13.41 ± 1.53^d^
	nonanal	Fat, citrus	1	14.19 ± 1.45^a^	6.79 ± 1.76^b^	4.04 ± 0.17^d^	6.01 ± 0.18^bc^	4.77 ± 0.27^cd^
	heptanal	Fat, fruity, dry fish	3	10.54 ± 0.22^a^	8.80 ± 0.73^b^	9.41 ± 0.65^b^	6.69 ± 0.24^c^	6.27 ± 0.25^c^
	(E)-2-heptenal-M	Fat, fruity	13	3.03 ± 0.18^a^	2.99 ± 0.06^a^	2.38 ± 0.26^b^	2.42 ± 0.03^b^	2.17 ± 0.13^b^
	propanal	Malty	15.1	2.00 ± 0.13^a^	0.84 ± 0.08^b^	0.66 ± 0.04^c^	0.83 ± 0.05^b^	0.83 ± 0.05^b^
	(E)-2-heptenal	Fat, fruity	13	2.30 ± 0.14^b^	1.63 ± 0.06^c^	1.34 ± 0.06^d^	1.48 ± 0.01^c^	1.48 ± 0.03^c^
	(E)-2-octenal	Nut, fat	11.41	1.92 ± 0.20^a^	1.62 ± 0.22^a^	1.77 ± 0.10^a^	1.77 ± 0.12^a^	1.73 ± 0.05^a^
	hexanal	Green, fresh grass	9.1	2.38 ± 0.13^a^	1.59 ± 0.11^b^	1.28 ± 0.06^c^	1.56 ± 0.03^b^	1.60 ± 0.09^b^
	(E)-2-hexenal	Green, fruity	17	2.32 ± 0.30^a^	1.44 ± 0.09^b^	1.03 ± 0.08^c^	0.64 ± 0.04^d^	0.82 ± 0.02^cd^
	3-methyl-2-butenal	Fruity	29.14	0.46 ± 0.03^d^	1.25 ± 0.05^a^	0.87 ± 0.10^c^	0.77 ± 0.03^c^	0.83 ± 0.04^c^
Alkenes	α-pinene	Woody, pine	6	0.95 ± 0.35^d^	8.91 ± 0.34^a^	6.72 ± 0.63^c^	7.17 ± 0.41^b^	6.54 ± 0.08^c^
	limonene	Citrus, mint	34	0.15 ± 0.01^e^	11.20 ± 0.04^a^	6.24 ± 0.01^d^	9.25 ± 0.01^c^	10.27 ± 0.00^b^
	β-myrcene-M	Herbal, woody	15	0.76 ± 0.06^d^	2.95 ± 0.21^a^	2.46 ± 0.21^b^	1.00 ± 0.08^cd^	1.16 ± 0.03^c^
	β-myrcene-D	Herbal, woody	15	0.16 ± 0.06^b^	1.33 ± 0.03^a^	1.10 ± 0.09^b^	0.25 ± 0.01^c^	0.19 ± 0.01^c^
	terpinolene	Pine	200	0.05 ± 0.01^a^	0.08 ± 0.01^a^	0.08 ± 0.01^a^	0.09 ± 0.01^a^	0.11 ± 0.01^a^
	β-pinene	Pine, resin	140	0.05 ± 0.01^a^	5.06 ± 0.00^a^	3.01 ± 0.00^a^	6.07 ± 0.00^a^	7.98 ± 0.00^a^
	camphene	Minty, citrus, green, spicy nuances.	880	0.08 ± 0.00	0.04 ± 0.00	0.04 ± 0.00	0.03 ± 0.00	0.03 ± 0.00
Esters	hexyl acetate-M	Citrus, sweet, pineapple, banana, pear	2	2.40 ± 0.22^e^	17.58 ± 2.52^a^	7.19 ± 0.11^d^	13.56 ± 0.35^b^	9.31 ± 0.27^c^
	methyl acetate	Berry, sweet, fruity	3	0.56 ± 0.17^d^	5.79 ± 0.09^a^	1.22 ± 0.24^c^	1.58 ± 0.07^b^	1.41 ± 0.10^b^
	lsoamyl acetate	Fruity, sweet	93.9	0.08 ± 0.01	0.02 ± 0.00	0.02 ± 0.00	0.03 ± 0.00	0.02 ± 0.00
	propyl butyrate	Banana, pineapple	18	0.62 ± 0.18^a^	0.47 ± 0.08^a^	0.46 ± 0.02^a^	0.60 ± 0.04^a^	0.50 ± 0.01^a^
	lsobutyl propionate	Fruity, sweet	100	0.53 ± 0.07^a^	0.24 ± 0.00^b^	0.17 ± 0.01^b^	0.24 ± 0.01^b^	0.24 ± 0.00^b^
	ethyl formate	Fruity, rum	6600	0.01 ± 0.00	ND	ND	ND	ND
	hexyl butyrate	Almond, fruity pineapple	1000	0.08 ± 0.00	0.04 ± 0.01	0.02 ± 0.00	0.03 ± 0.00	0.02 ± 0.00
	lsobutyl acetate	Floral, strawberry	922	0.05 ± 0.00	0.04 ± 0.00	0.03 ± 0.00	0.04 ± 0.00	0.04 ± 0.00
	butyl butanoate-D	Apple, pineapple	14,066	0.05 ± 0.00	ND	ND	ND	ND
	ethyl 3-hydroxybutyrate	Fruity	20,000	0.02 ± 0.00	0.01 ± 0.00	0.01 ± 0.00	0.01 ± 0.00	0.01 ± 0.00
Alcohols	tert-butanol	Camphor, fresh	1.8	1.46 ± 1.14^b^	3.15 ± 0.65^a^	1.07 ± 0.26^b^	3.12 ± 0.34^a^	3.73 ± 0.43^a^
	(Z)-3-hexen-1-ol	Grass	70	1.15 ± 0.22^d^	1.53 ± 0.53^cd^	1.92 ± 0.06^bc^	2.27 ± 0.06^ab^	2.59 ± 0.05^a^
	cis-2-Penten-1-ol	Grass, fruity	7.5	0.28 ± 0.04^d^	1.11 ± 0.07^a^	1.35 ± 0.17^a^	0.87 ± 0.09^b^	0.63 ± 0.04^c^
	butanol-D	Fruity, jasmine	480	2.71 ± 0.15^a^	0.26 ± 0.11^b^	0.21 ± 0.00^bc^	0.28 ± 0.00^b^	0.12 ± 0.01^c^
	2-heptanol	Fruity, herb	70	0.11 ± 0.02^a^	0.13 ± 0.01^b^	0.11 ± 0.00^c^	0.16 ± 0.00^c^	0.17 ± 0.01^bc^
	isobutanol	Alcoholic	33	1.21 ± 0.12^a^	0.54 ± 0.03^bc^	0.43 ± 0.04^c^	0.57 ± 0.04^b^	0.56 ± 0.01^b^
	2-hexanol-M	Floral, rose	250	0.62 ± 0.03	0.27 ± 0.01	0.23 ± 0.01	0.19 ± 0.01	0.19 ± 0.00
	2- hexanol-D	Floral, rose	250	0.02 ± 0.00	0.02 ± 0.00	0.02 ± 0.00	0.02 ± 0.00	0.02 ± 0.00
	3-methyl-1-butanol-M	Malty	250	0.24 ± 0.00	0.03 ± 0.00	0.05 ± 0.00	0.08 ± 0.01	0.04 ± 0.00
	isoprenol	Fruity, herb	123.2	0.43 ± 0.04^a^	0.06 ± 0.01^b^	0.03 ± 0.00^c^	0.04 ± 0.00^c^	0.05 ± 0.00^bc^
	isoprenol -M	Floral, green	1000	0.14 ± 0.01	0.06 ± 0.00	0.06 ± 0.00	0.06 ± 0.00	0.05 ± 0.00
	1-penten-3-ol	Fruity, mud	400	0.03 ± 0.00	0.02 ± 0.00	0.02 ± 0.00	0.03 ± 0.00	0.03 ± 0.00
	pentanol	Sweet, bread, grain	4000	0.02 ± 0.00	0.01 ± 0.00	0.01 ± 0.00	0.01 ± 0.00	0.01 ± 0.00
	isobutanol-D	Alcoholic	7000	0.04 ± 0.00	0.03 ± 0.00	0.02 ± 0.00	0.03 ± 0.00	0.03 ± 0.00
	butanol	Fruity, jasmine	480	0.04 ± 0.00	0.02 ± 0.00	0.02 ± 0.00	0.02 ± 0.00	0.02 ± 0.00
	1 -hexanol-D	Floral, green	1000	0.05 ± 0.00^a^	0.02 ± 0.00^b^	0.02 ± 0.00^b^	0.02 ± 0.00^b^	0.01 ± 0.00^b^
	ethanol	Alcoholic	100,000	< 0.01	<0.01	<0.01	<0.01	<0.01
	2,3-Butanediol	Butter	150,000	< 0.01	<0.01	<0.01	<0.01	<0.01
Pyrazines	2,5-dimethylpyrazine	Popcorn, toast	8	1.24 ± 0.12^c^	2.90 ± 0.31^b^	2.78 ± 0.12^b^	3.38 ± 0.30^a^	2.93 ± 0.08^b^
	2-methylpyrazine	Nut, toast	60	0.32 ± 0.07^a^	0.26 ± 0.03^ab^	0.21 ± 0.01^b^	0.25 ± 0.02^ab^	0.26 ± 0.00^a^
	pyrazine	Hazelnut	6000	<0.01	0.01 ± 0.00	0.01 ± 0.00	0.01 ± 0.00	0.01 ± 0.00

Note: A: Represents the flavor descriptions are obtained from the technology of food flavoring (Sun, 2017) and http://www.odour.org.uk B: Represents the aroma threshold of flavor compounds is mainly derived from the technology of food flavoring (Sun, 2017). Note: a, b, c, d and e Means with different letters within a row differ significantly (*P* < 0.05). ±: Represents the standard deviation. n = 3; The control sample was designated as “K”; Tsingtao Flower hop pellets soaked in boiling water was labeled “GK”; Tsingtao Flower hop pellets soaked in cold water was labeled “GL”; Saaz hop pellets soaked in boiling water was labeled “JK”; Saaz hop pellets soaked in cold water was labeled “JL”.

Ketones are crucial for bread baking and contribute to floral and fruity aromas. Their flavor profiles depend on molecular weight. Smaller ketones yield fruity aromas, while larger ketones present floral profiles. Ketones are primarily produced through the following pathways: (1) the Maillard reaction, (2) lipid oxidation, (3) chain ketone degradation, and (4) glycolysis and the Ehrlich pathway (An et al., 2023). Acetoin was the most significant contributor to bread flavor among the detected ketones. As a byproduct of glycolysis during sourdough fermentation, acetoin significantly enhances the floral aroma of bread owing to its low threshold and molecular weight. It is a typical byproduct of carbohydrate metabolism, produced exclusively in the presence of fermentable carbohydrates or pyruvic acid. Some yeast can also generate acetoin (Wang et al., 2021).

1-Octen-3-one, known for its potato aroma, showed ROAV values above 15 across all five samples, although its effect varied based on the type and quantity of hops. In the K sample, the ROAV for 1-octen-3-one was 18.85, with significant increases in samples containing hops. The GL and JL samples exhibited ROAVs exceeding 65, while GK and JK samples showed values above 45, though these were significantly lower than those of GL and JL (*P* < 0.05). This suggests that low- and high-temperature soaking methods significantly influence the flavor profile of this compound, with low-temperature soaking preserving a higher flavor intensity. As a carbonyl compound formed through lipid degradation, often from linoleic acid, 1-octen-3-one can interact with amino compounds, producing aldehyde amines or condense with alcohol, to form aldoses and nitrogen-containing polymers (Bleicher et al., 2022). These reactions under high-temperature soaking reduce ROAV values, while low-temperature soaking may mitigate these reactions and retain flavor intensity.

A similar trend was observed with 3-pentanone, which had an ROAV of 9.12 in the control bread but exceeded 10 in all samples containing hops, following the pattern of 1-octen-3-one. The highest ROAVs were recorded in the GL and JL samples, followed by GK and JK, with Saaz hops contributing higher flavor intensity than Tsingtao hops (*P* < 0.05), confirming that both the soaking method and hop type influence flavor strength, aligning with previous findings (Guimarães et al., 2021). 3-Pentanone, with its low molecular weight and threshold, imparts a strong fruity flavor to bread and it is primarily produced through fat oxidation. Conversely, 2-octanone has a high molecular weight and a pleasant floral aroma. Song et al. (2022) showed that 2-octanone significantly enhances the characteristic flavor of meat. The ROAV of 2-octanone exceeded 9, and its contribution to bread flavor increased significantly with the addition of hops. In the GL and GK samples, the ROAV values from cold water soaking were significantly higher than those from boiled water soaking, while the opposite trend was observed for JL and JK. 2-Octanone is formed during sourdough fermentation through the oxidation and hydrolysis of saturated fatty acids, and the addition of hops accelerates fat hydrolysis, enriching the fruity and herbal aroma of the bread. Although sulcatone and 2-heptanone exhibited fruity aromas, their contributions decreased with the addition of hops. This reduction varied by hop species and soaking method; cold water soaking preserved flavor better, and Tsingtao hops contributed more to the fruity aroma than Saaz hops (*P* < 0.05).

During baking, the Maillard reaction in the dough matrix produced furan and nitrogen‑sulfur compounds, imparting caramel, butter, and coffee aromas to the bread. Furans and sulfur were generated through Maillard reactions and lipid degradation. At high temperatures, organic acids react with alcohols to form esters and pyrazines. In the control (K) sample, 2-methylfuran, 2-pentylfuran, and ethylbenzene contributed significantly to the aroma (*P* < 0.05). The addition of hops decreased the levels of these compounds. 2-Methylfuran is typically produced from the thermal breakdown of linoleic acid hydroperoxides and esters. Hops may reduce hydroperoxide formation and lipid oxidation, which decreases the aromatic intensity of 2-methylfuran. The trends in flavor loss for these compounds were similar to 2-octanone. Flavor differences depend on the hop type and soaking method used. For Tsingtao hops, cold water soaking effectively preserved the characteristic flavor intensity of 2-methylfuran and 2-pentylfuran, while boiling water soaking significantly lowered their intensity (*P* < 0.05). In contrast, the opposite trend was observed for Saaz hops. These complex chemical reactions may be influenced by genetic factors, variety, harvesting practices, environmental conditions, and fertilizers (Dabbous-Wach et al., 2021; Féchir et al., 2023). After the addition of hops, 2-ethylfuran and diallyl sulfide, along with aromas of butter, burnt sugar, coffee, and garlic, showed a marked increase, with Saaz hops yielding the highest flavor intensity. Alkyl pyrazines are crucial products of the Maillard reaction, providing roasted, nutty, and meaty aromas reminiscent of popcorn. Among the three identified pyrazine compounds, only 2,5-dimethylpyrazine exhibits a ROAV >1, positively influencing product acceptability and consumer appetite (Scalone et al., 2020). The flavor contribution of 2,5-dimethylpyrazine significantly increased with hop addition (*P* < 0.05), with the highest enhancement in bread made with cold water-soaked hops, followed by boiled water-soaked hops.

Aldehydes are generated through the Maillard reaction or lipid degradation at elevated baking temperatures through reactions between reducing sugars and amino acids. Additionally, some aldehydes may form within yeast cells through the degradation of flour amino acids through the Ehrlich pathway (Sun et al., 2024). Ten aldehyde compounds were identified, with most showing a decrease in ROAV following hop addition. Only two aldehydes, 2-methyl butanal and 3-methyl-2-butenal, exhibited increased flavor intensity with hop addition. 2-Methyl butanal, known for its robust, malty flavor derived from isoleucine, correlates positively with bread aroma; higher concentrations enhance pleasantness and freshness for consumers (Chen et al., 2020). After hop addition, flavor contribution increased from 9.04 to 35.78 in GL and 19.09 in JL, mirroring the trend of ketones and significantly enhancing malty aroma. Cold water-soaked hops showed a significant advantage over boiled water-soaked hops (*P* < 0.05). 3-Methyl-2-butenal, characterized by its fruity aroma, is similarly influenced by soaking method and hop type, with cold water-soaked Tsingtao hops being more effective than boiled Saaz hops.

The addition of hops resulted in a decrease in eight aldehydes, including lipid oxidation products, such as nonanal, heptanal, and propanal. At low concentrations, these aldehydes contribute to malt and fruity flavors; however, at high concentrations, they produce undesirable fatty odors (Liu et al., 2022). Hops significantly reduced these off-flavor compounds, with Saaz hops proving particularly effective against lipid oxidation. Hexanal, another lipid oxidation product, exhibited a rancid and spicy flavor at elevated concentrations, which considered a typical off-flavor (Leonard et al., 2022). The addition of hops reduced the levels of these off-flavor compounds, with the GK sample showing the best performance. Fatty aldehydes primarily arise from lipid oxidation. In the absence of hops, the dough contained fewer yeast cells, leading to more available oxygen for lipid oxidases to produce a high quantity of aldehydes through iron oxidation. When hops are added, yeast growth promoted during fermentation and proofing consumes more oxygen, thereby reducing lipid oxidation. Additionally, high baking temperatures further decrease aldehyde synthesis by inactivating lipid oxidase activity (Sun et al., 2024).

Alkenes, which typically exhibit citrus, mint, green, and woody aromas, significantly increased in intensity with hop addition, particularly in the GL sample. Limonene, a natural monoterpene known for its antibacterial and antifungal properties, showed significant increases of 73.67-fold in GL and 67.47-fold in JL, making it a biomarker for hop-infused bread. α-Pinene, characterized by its woody aroma and potential neuroprotective effects, also exhibited an increased flavor contribution after hop addition, with the GL sample showing an 8.38-fold increase compared to the control. Despite its lower concentrations, α-pinene has a lower threshold, allowing it to contribute more significantly to bread aroma than the higher-concentration β-pinene. β-Myrcene, one of the most significant monoterpenes in hops, is noted for its herbal and woody aromas, but it is susceptible to degradation at high baking temperatures (Rettberg et al., 2018). Bread made with Tsingtao hops exhibited significantly higher β-myrcene flavor intensity than that of Saaz hops, possibly due to variations in growing conditions, climate, or genetic factors. Additionally, the soaking method is also an important factor. Kemp et al. (2021) reported that elevated temperatures accelerated the loss of hop volatiles in beer. Consistently, our results confirm that extraction temperature critically influences the retention of hop aroma compounds in bread. The distinct profile of cold-extracted Tsingtao hops may be attributed to the preservation of thermolabile mono- and sesquiterpenes, which are otherwise degraded at high soaking temperatures.

Esters are volatile flavor compounds associated with fruity, floral, and citrus aromas, playing a crucial role in fermentation aroma. In hop bread, some esters arise from the reaction between acetyl-CoA derivatives of fatty acids (C6–C10) and alcohols, catalyzed by acetyltransferases in yeast cells (Boto et al., 2025). Thirteen esters were identified, with only hexyl acetate-M and methyl acetate exhibiting an ROAV of >1. These compounds, which emit citrus, sweet, pineapple, and berry aromas, exhibited lower aroma intensities in bread lacking hops. However, the addition of hops significantly enhanced aroma contribution, with Tsingtao hops and cold-water soaking providing the greatest enhancement, particularly for the prominent hexyl acetate-M. Hong et al. (2022) reported that hop variety significantly modulates the accumulation of volatile aroma compounds during maturation. In particular, increased levels of terpenes and esters were associated with enhanced citrus and fruity sensory attributes in both hop tea and late-hopped beer.

Alcohols are the predominant flavor compounds in hop bread, typically imparting fruity aromas. Despite their high thresholds and low volatility, which complicate detection, alcohol significantly influences the overall flavor profile. Eighteen alcohol compounds were detected, with only five having ROAV values >1. Tert-butanol, (Z)-3-hexen-1-ol, and cis-2-Penten-1-ol, characterized by camphor, fresh, and grassy aromas, respectively, exhibited increased contributions after hop addition. Tert-butanol and (Z)-3-hexen-1-ol showed significant increases in the JK sample, while cis-2-Penten-1-ol significantly increased in GK. Conversely, soaking hops in boiled water enhanced their distinctive flavor profile, while other compounds did not benefit as much from this method. The aroma intensities of butanol-D and isobutanol significantly decreased, possibly due to the Ehrlich pathway, which converted amino acids into fusel aldehydes during sourdough fermentation. This process involves converting α-keto acids into fusel aldehydes. Lactic acid bacteria preferentially utilize amino acids, reducing the precursors available for higher alcohol synthesis and decreasing the synthesis efficiency of butanol-D and isobutanol. Additionally, lactic acid produced by lactic acid bacteria may suppress higher alcohol formation through metabolic interference (Wang et al., 2021).

#### 3.8.2 Analysis of combined OPLS-DA

**Fig. 5:**
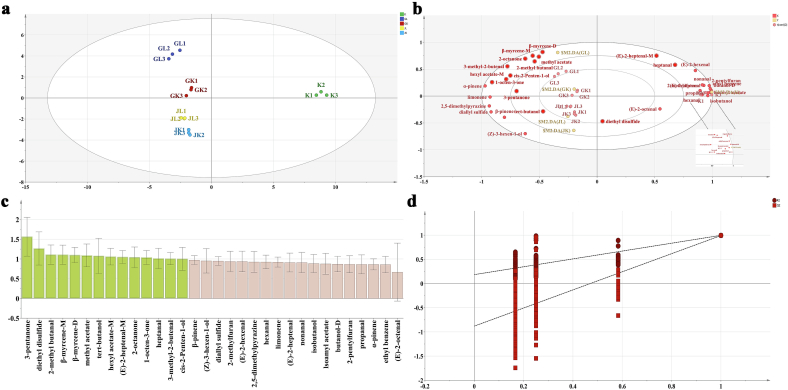
OPLS-DA model of volatile compounds from hop breads. Note: (a) Scores plot (b) Biplot parameter: R^2^X = 0.952, R^2^Y = 0. 980, Q^2^ = 0.938. Orange means category information, and green means volatile compounds (c) VIP scores. Green corresponds to compounds with VIP > 1, and pink represents compounds with VIP < 1. (d) cross-validation plot for the OPLS-DA model with 200 calculations in a permutation test: R^2^ = (0.0, 0.184), Q^2^ = (0.0, −0.880). (For interpretation of the references to color in this figure legend, the reader is referred to the web version of this article.)

PCA is as an unsupervised dimensionality reduction technique, while OPLS-DA is a supervised method that offers greater efficiency and accuracy in differentiating between samples. Fig. 5(a) and (b) present the score plot and biplot for the bread samples, respectively, showing non-overlapping samples that indicate distinct flavor profiles. Hop significantly modifies the characteristic flavor of the bread compared to the K sample. Fig. 5(b) illustrates the correlation between the samples and specific volatile compounds, with spatial proximity reflecting a stronger correlation. The K sample was strongly correlated with aldehydes and furans, specifically hexanal, propanal, 2-methylfuran, and 2-pentylfuran. The incorporation of hops enhanced the volatile profile of the samples by adding typical fermentation aromas, including ketones, esters, alcohols, and hop-specific monoterpenes (α-pinene, β-pinene, limonene, and β-myrcene-M). These compounds enhance fruity aromas and possess significant physiological functions. To identify compounds crucial for sample differentiation, variable importance in projection (VIP) values, with VIP > 1 signifying key compounds. Fig. 5(c) presents 14 VOCs with VIP > 1, including three ketones, four aldehydes, two esters, two alkenes, two alcohols, and one sulfur compound. Fig. 5(d) presents the OPLS-DA permutation test graph, indicating R^2^ = (0.0, 0.184) and Q^2^ = (0.0, −0.880). A negative Q^2^ value validates the stability and effectiveness of the model. Moreover, the interception of the Y-axis of the Q^2^ regression line below zero confirms a valid and dependable classification model without overfitting.

### 3.9 Analysis of correlation

**Fig. 6:**
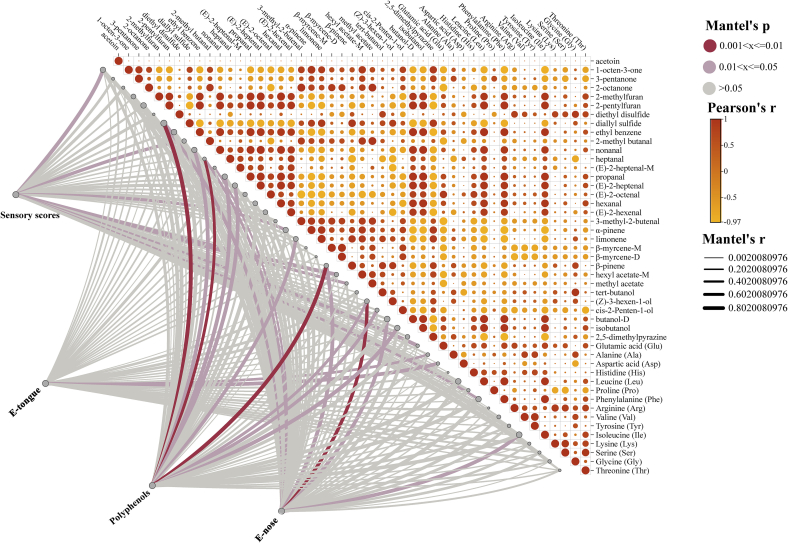
Analysis of the correlation between amino acids and volatile organic compounds.

Fig. 6 illustrates the correlation analysis among key volatile compounds, *E*-nose, E-tongue, amino acids, polyphenols, and sensory scores using Pearson's and Mantel's tests. The Mantel test is a robust statistical tool for assessing correlations between distance matrices, making it particularly valuable for multidimensional food flavor analysis (Mu et al., 2022). The findings show significant correlations among the compounds, *E*-nose, E-tongue, amino acids, polyphenols, and sensory scores, supported by Pearson and Mantel tests.

Pearson analysis revealed negative correlations between amino acids and most ketones (e.g., 1-octen-3-one, 3-pentanone, 2-octanone) and alkenes (e.g., α-pinene, limonene, β-myrcene-M). Conversely, certain furans—specifically 2-methylfuran, diethyl disulfide, and 2-pentylfuran—exhibited positive correlations with aldehydes, including nonanal, propanal, (E)-2-heptenal, (E)-2-octenal, and hexanal. These findings suggest that amino acids contribute to volatile formation through Maillard reactions, Strecker degradation, and lipid oxidation. Positive correlations with compounds such as hexanal and 2-pentylfuran, which are associated with caramelized and roasted notes, highlight their sensory relevance. However, elevated concentrations of these compounds may negatively affect flavor perception, as indicated by their inverse correlations with consumer acceptability (Franklin & Mitchell, 2019). In the Mantel test, the color and thickness of the lines represents the statistical significance and magnitude of Mantel's r-statistic, respectively. Red lines indicate highly significant correlations (Mantel's *P* < 0.01), while purple lines represent significant correlations (Mantel's *P* < 0.05), and gray lines indicate non-significance. Sensory scores correlated positively with 1-octen-3-one, 2-methylbutanal, α-pinene, hexyl acetate-M, and methyl acetate, underscoring their significance in favorable sensory evaluations (Mantel's *P* ≤ 0.01). Furthermore, the sensory scores significantly correlated with NMS (glutamic acid) and bitter (isoleucine) amino acids, underscoring the importance of these compounds in flavor perception. The E-tongue showed significant correlations with amino acids, including alanine (Mantel's *P* = 0.04, Mantel's *r* = 0.51) and aspartic acid (Mantel's *P* = 0.03, Mantel's *r* = 0.79), indicating their critical roles in taste perception. The E-nose strongly correlated with diallyl sulfide, heptanal, (E)-2-heptenal-M, β-pinene, and butanol-D. Among these, (Z)-3-hexen-1-ol exhibited the strongest correlation with the E-nose (Mantel's *P* = 0.008, Mantel's *r* = 0.70), consistent with the ROAV and OPLS-DA analyses. These findings confirm the effectiveness of E-nose and E-tongue technologies in detecting key flavors and taste attributes of bread.

Additionally, polyphenols exhibited strong correlations with volatile compounds. The Mantel test indicated significant associations between polyphenols and 10 key volatiles, particularly diallyl sulfide, heptanal, and β-pinene (Mantel's *P* ≤ 0.01). This underscores the role of polyphenols in modulating lipid oxidation and influencing the volatile profiles of the hop bread samples. Liu et al. (2023) reported that exogenous polyphenols modulate aroma profiles in heat-sterilized bayberry juice by inhibiting lipid oxidation and suppressing the formation of off-flavor compounds.

## 4 Conclusion

This study comprehensively examined the effects of various soaking methods (cold and boiled) and hop varieties (Tsingtao and Saaz) on the flavor profiles, sensory properties, and polyphenol contents of hop-infused sourdough breads. The evaluation of VOCs, polyphenols, amino acids, and sensory attributes in the samples was conducted using HS-GC-IMS, bionic sensing, UPLC-Orbitrap-MS, and automatic amino acid analysis. In total, 71 VOCs and 39 polyphenols were identified. Using ROAV combined with OPLS-DA, 14 key VOCs were identified, including three ketones, four aldehydes, two esters, two alkenes, two alcohols, and one sulfur. Cold-soaked Tsingtao hops yielded the highest sensory scores, attributed to their different volatile profile, particularly the presence of ketones and aldehydes, which significantly enhanced the aroma of the bread. Conversely, Saaz hops, especially after boiling, exhibited a higher concentration of polyphenols, contributing to greater SCS and antioxidant properties. However, this reduced sensory appeal compared to the cold-soaked Tsingtao hops. The hop varieties exhibited the following significant differences: Tsingtao hops enhanced aroma and overall flavor more effectively, while Saaz hops, despite their richness in bioactive compounds, primarily contributed to bitterness and astringency. This study establishes a foundation for optimizing hop variety selection and soaking methods in bread production.

## CRediT authorship contribution statement

**Chunyuan Ping:** Writing – review & editing, Writing – original draft, Data curation, Conceptualization. **Bian Li:** Validation, Supervision. **Yueyue Gao:** Project administration. **Xiang Li:** Visualization. **Fu Wang:** Resources, Project administration.

## Ethical statement

We ensured that participation was entirely voluntary, with no coercion involved. The national laws do not require ethical approval for sensory evaluation. There are no human ethics committees or formal documentation procedures available for sensory evaluation. Comprehensive information about the study's requirements and potential risks was fully disclosed to all participants. Their written consent was obtained before the commencement of the study. We pledged not to release any participant's data without their prior knowledge and consent. Furthermore, participants were given the liberty to withdraw from the study at any point they wished.

## Declaration of competing interest

The authors declare that they have no known competing financial interests or personal relationships that could have appeared to influence the work reported in this paper.

## Data Availability

Data will be made available on request.

## Appendix A Supplementary data

Supplementary material. Image 72
