# Supplementary material for: Exploring the mechanisms of flavor formation and polyphenolic changes in hop-infused sourdough bread affected by hop varieties and soaking methods
Source: Food Chem X. 2025 May 2;28:102512. doi: 10.1016/j.fochx.2025.102512 (PMC12139236; doi:10.1016/j.fochx.2025.102512)
Supplement: Supplementary file 1 — Supplementary material. [file mmc1.docx]

| Tabel S1  The sensory evaluation criteria of *Linjiangsi* broad bean paste | | |
| --- | --- | --- |
| Factors | Sensory standard | Scores |
| Appearance | Reddish-brown color, with a bright luster. | 10~7 |
|  | Deep-Yellow in color, with poor brightness. | 6~4 |
|  | Dull in color, without any brightness. | 3~1 |
| Aroma | Strong fruity, floral, pineapple, nutty, roast flavor. | 10~7 |
|  | Insufficient Strong fruity, floral, pineapple, nutty, roast flavor. | 6~4 |
|  | Lacks some distinctive flavors, only salty and bitter. | 3~1 |
| Taste | Strong umami, spicy and savory flavors. | 10~7 |
|  | Lacks of umami, spicy and savory. | 6~4 |
|  | Overly salty, without any umami taste. | 3~1 |
| Texture | Moderately thick, no visible impurities, soft and sticky broad beans. | 10~7 |
|  | Sauce is slightly thin with some impurities, broad beans are slightly hard. | 6~4 |
|  | The texture of sauce is thin, with more dusts. | 3~1 |

| Tabel S2  Characteristics and Performance Parameters of E-nose Sensor Array | | | |
| --- | --- | --- | --- |
| Sensors | Performance Description | Sensors | Performance Description |
| P10/1 | Sensitive to non-polar compounds (octane) | LY2/LG | Sensitive to strongly oxidizing gases (sulfides) |
| P10/2 | Sensitive to non-polar flammable gases (methane, heptane) | LY2/G | Sensitive to toxic gases (methylamines) |
| P40/1 | Sensitive to strongly oxidizing gases (methyl furfuryl disulfide) | LY2/AA | Sensitive to organic compounds (ethanol, acetone, ammonia) |
| PA/2 | Sensitive to organic compounds (acetaldehyde,) | LY2/Gh | Sensitive to toxic gases (anilines) |
| P30/1 | Sensitive to flammable organic compounds (ethanol) | LY2/gCTI | Sensitive to toxic gases (sulfides) |
| P40/2 | Sensitive to strongly oxidizing gases (methanethiol) | LY2/gCT | Sensitive to flammable gases (butane, propane) |
| P30/2 | Sensitive to organic compounds (hydrogen sulfide, ketones) | T40/2 | Sensitive to strongly oxidizing gases (dimethyl disulfide) |
| T30/1 | Sensitive to polar compounds (propanol, butane) | T40/1 | Sensitive to strongly oxidizing gases (creosote) |
| T70/2 | Sensitive to aromatic compounds (toluene and its derivatives) | TA/2 | Sensitive to organic compounds (hexanol) |

| Tabel S3  Sensory scores of *hops bread* soaked by cold and boiled water | | | | | | | |
| --- | --- | --- | --- | --- | --- | --- | --- |
| Sample | Objectives | | Appearance | Aroma | Taste | Texture | Sum |
| K | 1 | | 8.5 | 7.1 | 9 | 8.1 | 32.9 |
|  | 2 | | 7.3 | 8.5 | 8.8 | 8.2 |  |
|  | 3 | | 8.8 | 8.3 | 8.5 | 8.2 |  |
|  | 4 | | 8.3 | 7.8 | 8.3 | 7.4 |  |
|  | 5 | | 8.2 | 7.3 | 7.6 | 9 |  |
|  | 6 | | 7.3 | 8.2 | 8.6 | 8.5 |  |
|  | 7 | | 9 | 8.3 | 8.6 | 7.7 |  |
|  | 8 | | 7.9 | 8 | 7.7 | 8.5 |  |
|  | 9 | | 8.7 | 8.8 | 8.8 | 8.3 |  |
|  | 10 | | 8.4 | 9 | 8.4 | 7.1 |  |
| Average | | | 8.24 | 8.13 | 8.43 | 8.10 |  |
| GL | 1 | | 9.2 | 9.8 | 9.3 | 9.7 | 37.26 |
|  | 2 | | 9.8 | 9.2 | 9.8 | 8.4 |  |
|  | 3 | | 9.2 | 9.3 | 9.2 | 9.4 |  |
|  | 4 | | 9.1 | 9.8 | 9.8 | 9.8 |  |
|  | 5 | | 9.1 | 9.5 | 8.7 | 9.5 |  |
|  | 6 | | 9 | 9.3 | 8.5 | 8.7 |  |
|  | 7 | | 9.3 | 9.8 | 9.6 | 9.2 |  |
|  | 8 | | 8.9 | 9.3 | 9.9 | 8.7 |  |
|  | 9 | | 9.4 | 9.4 | 9.3 | 9.3 |  |
|  | 10 | | 8.8 | 9.3 | 9.5 | 9.8 |  |
| Average | | | 9.18 | 9.47 | 9.36 | 9.25 |  |
| GK | 1 | | 9.8 | 9.8 | 8.9 | 9.1 | 34.9 |
|  | 2 | | 9.5 | 9.5 | 8.5 | 8.9 |  |
|  | 3 | | 9.2 | 9.2 | 8.4 | 9.3 |  |
|  | 4 | | 9.3 | 9.3 | 8.4 | 9.1 |  |
|  | 5 | | 9.2 | 9.2 | 7.8 | 8.7 |  |
|  | 6 | | 9.3 | 9.3 | 8.6 | 8.6 |  |
|  | 7 | | 9.4 | 9.4 | 8.9 | 8.3 |  |
|  | 8 | | 8.4 | 8.4 | 7.7 | 9.1 |  |
|  | 9 | | 8.6 | 8.6 | 9.4 | 7.4 |  |
|  | 10 | | 8.1 | 8.1 | 8.8 | 8.6 |  |
| Average | | | 8.57 | 9.08 | 8.54 | 8.71 |  |
| JL | 1 | | 8.4 | 9.8 | 9.1 | 8.6 | 35.96 |
|  | 2 | | 8.8 | 9.2 | 9.2 | 9.2 |  |
|  | 3 | | 9.6 | 9.4 | 8.6 | 9.3 |  |
|  | 4 | | 9.3 | 8.1 | 8.6 | 8.5 |  |
|  | 5 | | 8.9 | 8.5 | 8.8 | 9.1 |  |
|  | 6 | | 9.3 | 8.3 | 8.5 | 8.3 |  |
|  | 7 | | 9.6 | 9.1 | 8.8 | 9.3 |  |
|  | 8 | | 8.9 | 9.3 | 9.5 | 9 |  |
|  | 9 | | 9.1 | 9.4 | 9.8 | 7.6 |  |
|  | 10 | | 8.8 | 9.4 | 9.4 | 9.2 |  |
| Average | | | 9.07 | 9.05 | 9.03 | 8.81 |  |
| JK | | 1 | 8.5 | 9.5 | 9.2 | 7.3 | 34.0 |
|  |  | 2 | 8.3 | 8.9 | 9 | 9 |  |
|  |  | 3 | 8.8 | 8.1 | 8.1 | 8.3 |  |
|  |  | 4 | 9.4 | 8.1 | 8.3 | 7.3 |  |
|  |  | 5 | 8.2 | 9 | 8.2 | 9.2 |  |
|  |  | 6 | 8.3 | 8.5 | 8.3 | 8.3 |  |
|  |  | 7 | 7.9 | 8.7 | 8.7 | 7 |  |
|  |  | 8 | 7 | 9.3 | 8.1 | 9 |  |
|  |  | 9 | 8.2 | 9.1 | 8.4 | 9 |  |
|  |  | 10 | 8.4 | 9.3 | 8.4 | 9.4 |  |
| Average | | | 8.3 | 8.85 | 8.47 | 8.38 |  |
